# Does *Calypogeia azurea* (Calypogeiaceae, Marchantiophyta) occur outside Europe? Molecular and morphological evidence

**DOI:** 10.1371/journal.pone.0204561

**Published:** 2018-10-10

**Authors:** Katarzyna Buczkowska, Vadim Bakalin, Alina Bączkiewicz, Blanka Aguero, Patrycja Gonera, Monika Ślipiko, Monika Szczecińska, Jakub Sawicki

**Affiliations:** 1 Department of Genetics, Faculty of Biology, Adam Mickiewicz University in Poznań, Poznań, Poland; 2 Botanical Garden-Institute, Russian Academy of Science, Vladivostok, Russia; 3 Duke Herbarium, Department of Biology, Duke University, Durham, NC, United States of America; 4 Department of Botany and Nature Protection, University of Warmia and Mazury in Olsztyn, Olsztyn, Poland; Universita degli Studi del Piemonte Orientale Amedeo Avogadro, ITALY

## Abstract

Oil bodies are the unique feature of most liverworts. Their shape, color and distribution pattern in leaf and underleaf cells are important taxonomic features of the genus *Calypogeia*. Most species of the genus *Calypogeia* have pellucid and colorless oil bodies, whereas colored, including gray to pale brown, purple-brown or blue oil bodies, are rare. To date, *C*. *azurea* was the only species with blue oil bodies to have been considered as a species of the Holarctic range. This species has been noted in various parts of the northern hemisphere–from North America, through Europe to the Far East. The aim of this study was to determine the genetic diversity of *C*. *azurea* from different parts of its distribution range and to ascertain whether blue oil bodies appeared once or several times in the evolution of the genus *Calypogeia*. The phylogenetic analyses based on four plastid regions (*rbcL*, *trnG*, *trnL*, *trnH-psbA*) and one nuclear region (ITS2) revealed that *C*. *azurea* is presently a paraphyletic taxon, with other *Calypogeia* species nested among *C*. *azurea* accessions that were clustered into four different clades. Based on the level of genetic divergence (1.03–2.17%) and the observed morphological, ecological and geographical differences, the evaluated clades could be regarded as previously unrecognized species. Four species were identified: *C*. *azurea* Stotler & Crotz (a European species corresponding to the holotype), two new species from Pacific Asia—*C*. *orientalis* Buczkowska & Bakalin and *C*. *sinensis* Bakalin & Buczkowska, and a North American species which, due to the lack of identifiable morphological features, must be regarded as the cryptic species of *C*. *azurea* with a provisional name of *C*. *azurea* species NA.

## Introduction

The genus *Calypogeia* (Raddi) comprises around 90 described species of leafy liverworts of the suborder Jungermanniineae [1, 2]. Oil bodies, intracellular organelles surrounded by a single unit membrane, are a unique feature of most liverworts [3]. Their shape, color and distribution pattern in leaf and underleaf cells are important taxonomic features of many liverwort species, including the species of the genus *Calypogeia*. Despite the above, oil bodies are not commonly used in taxonomic identification because they are absent in dried herbarium material [1, 4, 5]. Most species of the genus *Calypogeia* with identified oil bodies have pellucid and colorless oil bodies, whereas colored, including gray to pale brown, purple-brown or blue oil bodies, are rare [1, 6]. Gray to pale brown oil bodies were detected in *C*. *contracta* Inoue, purple-brown oil bodies were noted in *C*. *oblata* Herz., whereas deep blue oil bodies were found in five species, i.e. *C*. *aeruginosa* Mitten (intergradation between purple and blue), *C*. *goebelii* (Schiffn.) Steph., *C*. *granulata* Inoue (intergradations of brown within a single leaf), *C*. *lunata* Mitt., *C*. *peruviana* Ness & Mont., and *C*. *azurea* Stotler & Crotz [1, 7, 8, 9]. According to [10, 11], *Calypogeia* species contain azulene, a natural chromophore whose chemical structure is responsible for the dark blue color of oil bodies. Chemical analyses of *C*. *azurea* and *C*. *granulata* revealed the presence of 1,4-dimethylazulene and other azulenoids in live plants, but these compounds were not found in species with colorless oil bodies–*C*. *fissa* (L.) Raddi and *C*. *suecica* (Arnell & J.Perss.) Mull.Frib. [12–15].

In the group of species with blue oil bodies, *C*. *azurea* is the only widely distributed taxon throughout the northern hemisphere. *Calypogeia azurea* is regarded as a hemiboreal-montane species. The discussed taxon has been reported from North America, throughout Europe to East Asia, from various habitats ranging from loamy soil, humus, peat, wet stones and rocks to rotten logs [1, 16–20]. *Calypogeia* species are characterized by high environmentally induced variations in morphology [1], and live plants, including *C*. *azurea*, are identified based on the presence of blue oil bodies. *Calypogeia azurea* is the only species with blue oil bodies in Europe, where it occurs mainly in the mountains between low altitude regions to the Alpine belt [17, 19]. Two species with blue oil bodies have been identified in North America, *C*. *azurea* and *C*. *peruviana*, but they differ in distribution range. *Calypogeia azurea* is rare, and it occurs mainly in the Pacific Northwest, whereas *C*. *peruviana* is found in the southeastern United States from the Carolinas and east Tennessee to Florida and west to Mississippi. In Central and South America, the neotropical species *C*. *peruviana* is widespread, while *C*. *azurea* is not reported from this region. [1, 6, 21].

In Pacific Asia, blue oil bodies have been identified in *C*. *azurea* as well as in *C*. *aeruginosa*, *C*. *lunata* and *C*. *granulata*, but the three latter species clearly differ in the shape of leaves and underleaves and in the structure of the oil bodies [7, 9, 22]. In Asia, *C*. *azurea* is restricted to the oroboreal belt of mountain zone, south boreal, hemiboreal and cool temperate zones, and it does not occur in the far north [20, 23–24]. Dried specimens, in which oil bodies are absent, may be sometimes mistaken for other *Calypogeia* species, especially *C*. *muelleriana* (Schiffn.) Müll.Frib., *C*. *fissa*, *C*. *tosana* or *C*. *peruviana* [1, 17, 25].

Molecular tools, including fast-growing DNA barcoding proposed by Hebert *et al*. [26], open new possibilities for identifying plant species based on species-specific differences in short DNA sequences. DNA barcoding is also used to detect genetic diversity and discover new species [27]. Studies where molecular markers were applied to analyze liverwort taxonomy revealed that a morphology-based approach supports only partial detection of species diversity [28–33]. Numerous authors have demonstrated that the diversity of liverworts species is underestimated due to hidden genetic diversity [34]. Unrecognized species were discovered especially among liverwort taxa with a world-wide distribution, including *Frullania tamarisci* [29], *Metzgeria furcata* and *M*. *conjugata* [35], *Ptilidium pulcherrimum* and *P*. *ciliare* [36], *Porella platyphylla* [30], *Cololejeunea lanciloba* [37] and *Aneura pinguis* [38–39]. Genetic diversity was also found in *Calypogeia* species, including *C*. *fissa* [40], *C*. *muelleriana* [41–42] and *C*. *sphagnicola* [43–44].

The distribution of *C*. *azurea* in Holarctic region, its high morphological variability [1, 17], and the results of preliminary population genetic studies [45] support the hypothesis that *C*. *azurea* is not genetically homogeneous, but exhibits genetic diversity within its geographical range. In the present study, the DNA sequences of four plastid regions (*rbcL*, *trnG*, *trnL*, *trnH-psbA*) and one nuclear region (ITS2) were used to assess the genetic diversity of *C*. *azurea* from different parts of its distribution range. The study also attempted to determine whether blue oil bodies appeared once or several times during the evolution of the genus *Calypogeia*.

## Materials and methods

### Ethics statement

The collection of the material in Vietnam was carried out in accordance with obtained permission for collecting material and transferring of material across national borders provided by the Institute of Ecology and Biological Resources of VAST. The collection of the material in China was accomplished in the course of joint investigation within collaboration project of the Russian Foundation for Basic Researches and Chinese Foundation for Natural Sciences in accordance with permissions for collecting material from the Chinese province of Guizhou and for transfer of the living material across national borders. Suitable permission was also obtained for colleting materials in America. The Polish samples from Tatra and Bieszczady National Parks were collected with the permission given by Ministry of Environment, Department of Nature Conservation and the Directors of these National Parks. For the remaining locations specific permission was not required because species of the genus *Calypogeia* are neither endangered nor protected species.

### Plant material

**Fig 1:**
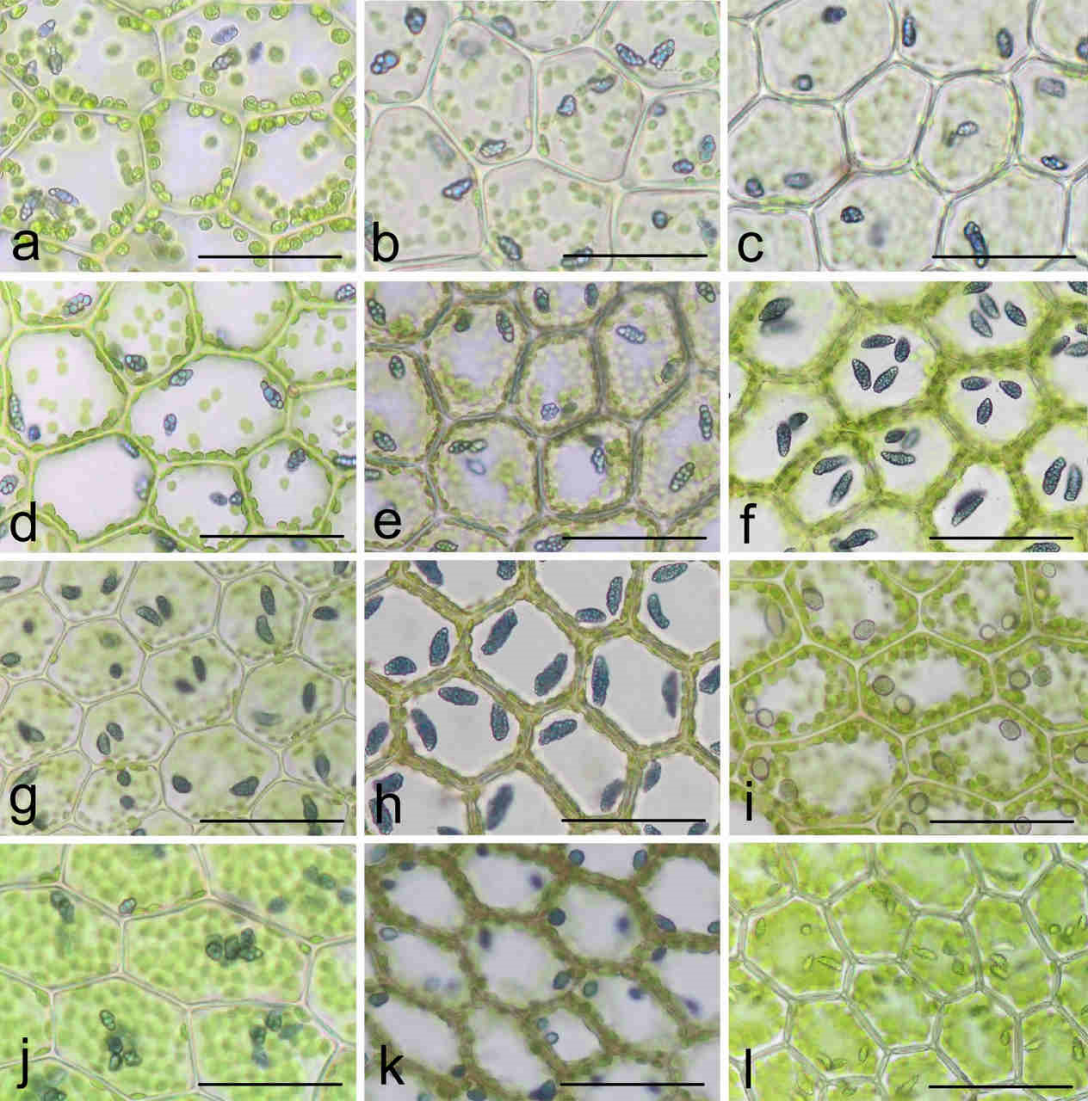
Cells of the *Calypogeia azurea* complex and the related taxa with oil bodies. (a) *C*. *azurea* s. str. (European clade; POZW 41390); (b-c) *C*. *azurea*—NA (North American clade; POZW 42444); (d-e) *C*. *orientalis* (VBGI P-39-4-15, VBGI P-8-1-16); (f-g) *C*. *sinensis* (China-56-78-13, China-56-77-13); (h-i) *C*. *granulata* (VBGI V-2-73-16, VBGI China 56-19-13); (j) *C*. *peruviana* (POZW 42619); (k) *C*. *aeruginosa* (VBGI V-8-75-17); (l) *C*. *tosana* (VBGI China-56-86-13). Bar = 50 μm.

A total of 40 samples initially identified as *C*. *azurea* were examined. Oil bodies were determined in 32 samples of live plants (Fig 1), and they were not identified in 8 herbarium specimens. The analyzed specimens originated from different regions of Europe, North America and Asia. Most samples were collected by authors, and several specimens were obtained from herbarium collections (S1 Table). The specimens of *C*. *azurea* were evaluated in molecular studies together with other *Calypogeia* species harboring blue oil bodies, i.e. *C*. *peruviana*, *C*. *granulata*, *C*. *aeruginosa* and *C*. *lunata*, as well as with *C*. *tosana* which has colorless oil bodies, but can be easily confused with *C*. *azurea*. Most of the Holarctic species with colorless oil bodies, i.e. *C*. *muelleriana*, *C*. *integristipula* Steph., *C*. *neesiana* (Massal. & Carestia) Mull.Frib., *C*. *suecica*, *C*. *fissa* and *C*. *sphagnicola* (Arnell & J.Perss.) Warnst. & Loeske, were used in phylogenetic analyses (Fig 1 and S1 Table).

Two species (*Calypogeia arguta* Ness & Mont and *C*. *sullivantii* Austin) from the subgenus *Asperifolia* were used as an outgroup based on the phylogeny described by Schuster [6]. Although *Asperifolia* is presently classified as a subgenus of *Calypogeia*, it is sufficiently genetically distinct to be used as an outgroup for the analyzed species of the subgenus *Calypogeia*. The sequences of the five studied DNA regions were newly generated for 39–73 specimens, depending on the region. Four DNA sequences of *C*. *azurea* were obtained from GenBank, and partial sequences of the remaining species were obtained from our previous studies (GB accession numbers are listed in S1 Table).

### DNA analysis

#### DNA extraction

Total genomic DNA was extracted from fresh or dried plants. In fresh samples, several stems from one specimen were ground with steel beads in a Bioprep-24 Homogenizer for 35 seconds, and DNA was extracted using the GeneJET Plant Genomic DNA Purification Mini Kit (Thermo Scientific) according to the manufacturer’s protocol. The Novabeads Plant DNA Kit (Novazym, Poland) was used to extract DNA from herbarium samples. The isolated DNA was dissolved in TE buffer and stored at –20°C. The quality of the isolated DNA was evaluated by electrophoresis in 0.8% agarose gel. The concentration and purity of DNA samples were determined using the Epoch Multi-Volume Spectrophotometer System.

#### PCR amplification and sequencing

Five DNA regions, including four chloroplast loci (*rbcL*, *trnL*, *trnG* and *trnH-psbA*) and one nuclear ITS2 region, were sequenced. The standard barcode region [46] was amplified for *rbcL* [47]. For *trnL* and *trnG*, introns were amplified according to Pacak & Szweykowska-Kulińska [48], and intergenic spacer *trnH-psbA* was amplified according to Sang *et al*. [49]. The internal transcribed spacer region ITS2 was amplified with the primers described by Sawicki *et al*. [50]. The same primers were used for the amplification and sequencing (S2 Table). PCR amplification was performed in a total volume of 20 μL, containing 2 μL of 10× PCR buffer with Mg^2+^ (Novazym; 25 mmol MgCl_2_), 1 μL of bovine serum albumin (0.25 mg/ml), 200 μmol of each dNTP (Novazym), 0.4 μmol of each primer, 1 U of Taq DNA polymerase (Novazym) and 1 μL of template DNA (about 40 ng). The PCR reaction was performed in the following cycling steps: 4 min of initial denaturation at 94ºC, followed by 30 cycles of 60 s at 94ºC, 30 s at annealing temperature (46–60 ºC, depending on the primer, see S2 Table) and 60 s at 72ºC, with a final extension step of 7 min of 72ºC. Finally, 5 μl of the amplification product was visualized on 1.0% agarose gel by GelView staining, and the remainder was purified with Exo-AP (Thermo Scientific) to remove unincorporated primers and dNTPs. Purified PCR products of the studied DNA regions were sequenced in both directions using the ABI BigDye 3.1 Terminator Cycle Kit (Applied Biosystems) and were visualized with the ABI Prism 3130 Automated DNA Sequencer (Applied Biosystems).

#### Data analysis

Chromatograms of DNA sequences were edited and assembled in Geneious R6 (Biomatters, USA). The assembled sequences were aligned with the Muscle algorithm [51] in MEGA 7 [52] using default settings. Regions of ambiguous alignment and incomplete data were excluded from the analysis, and lacking sequences were coded as missing. The differences between the examined specimens were illustrated with the neighbor-joining (NJ) method based on Kimura 2-parametr model [53] for individual and combined DNA regions. Next, phylogenetic trees were generated by maximum likelihood (ML) and maximum parsimony (MP) methods in MEGA 7. First, the analyses were performed for all samples, including samples with incomplete sequences. The analysis was then limited to the accessions with complete sequences of all studied DNA regions. This approach supported evaluations of both old samples that were difficult to sequence as well as GeneBank accessions with missing sequences in some of the analyzed loci. At the beginning, plastid and nuclear loci were analyzed separately. The possible conflicts between the plastid and nuclear data sets were detected in the incongruence length difference (ILD) test [54] with a custom ILD script (http://rossmounce.co.uk/) in TNT 1.1 [55–56] with 1000 replicates and default settings for the New Technology Search options. The script is available at http://phylo.wikidot.com/tntwiki#TNT_scripts. We tested for incongruence between data partitions within the chloroplast (4 partitions) and between combined chloroplast and nuclear data sets. The ILD test was run without an outgroup.

In maximum likelihood estimation, the best evolution model for each data set was determined using maximum likelihood model testing and the Bayesian Information Criterion (BIC) in MEGA 7, were discrete gamma distribution was modeled in four categories. The best models for combined plastid and ITS2 data were T92+G and T92, respectively. Maximum Parsimony analyses involved the following tree inference options: Tree-Bisection-Reconnection (TBR) as a search method with 10 number of initial trees (random-addition), search level of 3 and the maximum number of 100 trees to be retained in each step. The confidence of clades within the inferred trees was evaluated by the bootstrap method [57] with 1000 replicates. Genetic distance K2P for the pairs of sequences between and within the studied species was calculated to estimate the degree of evolutionary divergence.

The Automatic Barcode Gap Discovery (ABGD) software was used to group the examined specimens and identify potential hidden species within *C*. *azurea* based on pairwise distances by detecting differences between the intraspecific and interspecific variation (barcoding gap) without an *a priori* species hypothesis. The above program automatically detects the distance where the barcode gap is located, and it can even be used when intraspecific and interspecific distributions overlap to partition the data set into candidate species [58]. The ABGD analyses were performed on a web interface (http://wwwabi.snv.jussieu.fr/public/abgd/abgdweb.html) using all available distance metrics: JC69 [59], K2P and the uncorrected p-distance. Default settings of P value (Pmin = 0.001, Pmax = 0.1) and relative gap width X = 1.5 were used. ABGD relies on a two-steps process. In the first step, the sequences are divided into ABGD units based on a statistically inferred barcode gap as initial partitioning, and in the second step, the same procedure is applied to the groups obtained in the first step to create a recursive partition.

Haplotype networks with the median joining (MJ) option were calculated to examine the variations in the studied *C*. *azurea* accessions [60]. The MP option [61] was applied to identify redundant median vectors and links. Haplotype networks were developed in Network 5.0 (Fluxus Technology). Haplotypes were colored according to their geographical origin.

### Morphological analysis

The evaluated specimens of the *C*. *azurea* complex and the related taxa (S1 Table) were subjected to a morphological analysis. Five stems were collected for analysis from each specimen. Leaves and underleaves were isolated under the Olympus SZX16 dissecting microscope, and digital photographs were taken with the Olympus CX31 translucent microscope with the UC30 camera. Digital photographs were analyzed in Cell D3 Imaging Software (Olympus) to determine morphological variations. In each stem, every trait was measured at least 10 times. Microscopic images of oil bodies were captured in the identified evolutionary lineages of *C*. *azurea* (C. *azurea* s. str., *C*. *azurea–*NA, *C*. *sinensis*, *C*. *orientalis*) and the related taxa. In addition, drawings of gametophytes were made for the newly described species. The new species were described based on fresh specimens with oil bodies. The mean, minimum and maximum values of 47 quantitative traits [62] were computed in STATISTICA 12 (StatSoft. Inc.).

## Results

### The characteristics of chloroplast and nuclear sequences and incongruence tests

Four plastid regions (*rbcL*, *trnL*, *trnG*, *trnH-psbA*) and one nuclear region (ITS2) were sequenced. A total of 352 sequences were used in the analysis, including 286 previously unpublished sequences for ingroup and 18 sequences for outgroup. Four sequences were obtained from GenBank and 44 –from our previous studies. DNA sequences revealed that 6 herbarium specimens which were identified as *C*. *azurea* without verifying of oil bodies had been misidentified (S1 Table). These specimens were excluded from further analyses. The total alignment of the examined chloroplast regions in the studied *Calypogeia* species comprised 1814 bp, including 86 variable and 81 parsimony informative sites. The ILD test revealed no conflict between four chloroplast data sets (p = 0.563). The nuclear ITS2 region comprised 513 bp, including 33 variable and 30 parsimony informative sites. The length of the analyzed DNA regions in the studied species, including the numbers of variable and parsimony informative sites, is presented in Table 1. Non-coding loci were more informative. The most parsimony informative locus was *trnH-psbA* (7.56%) and the least parsimony informative locus was *rbcL* (3.31%).

**Table 1:** The length (in bp) of the analyzed DNA regions in units detected within the *C*. *azurea* complex and the related taxa.

Species	*rbcL*	*trnG*	*trnL*	*trnL*^1^	*trnH-psbA*	cp all	ITS2
*C*. *azurea* European clade	635	658	295	295	225	1813	505–507
*C*. *azurea* North American clade	635	649–656	295	295	225	1804–1811	480–500
*C*. *sinensis*	635	656	295	295	225	1811	499–501
*C*. *orientalis*	635	656–657	295	295	225	1811–1812	498
*C*. *peruviana*	635	658	295	295	225	1813	498–501
*C*. *granulata*	635	656	295	295	225	1811	496
*C*. *tosana*	635	656	295	295	225	1811	-
*C*. *aeruginosa*	-	-	295	-	-	-	-
*C*. *lunata*	-	-	295	-	-	-	-
Alignment length	635	659	295	295	225	1814	513
Conserved sites	614	623	271	271	206	1727	475
Variable sites (V)	21	35	24	16	19	86	33
Parsi-info sites (P)	21	32	18	16	17	81	30
% Parsi-info	3.31%	4.86%	6.10%	5.42%	7.56%	4.47%	5.85%
Singleton sites (S)	0	3	6	0	2	5	3

^1^The data set with *C*. *aeruginosa* and *C*. *lunata*.

### Phylogenetic analysis

**Fig 2:**
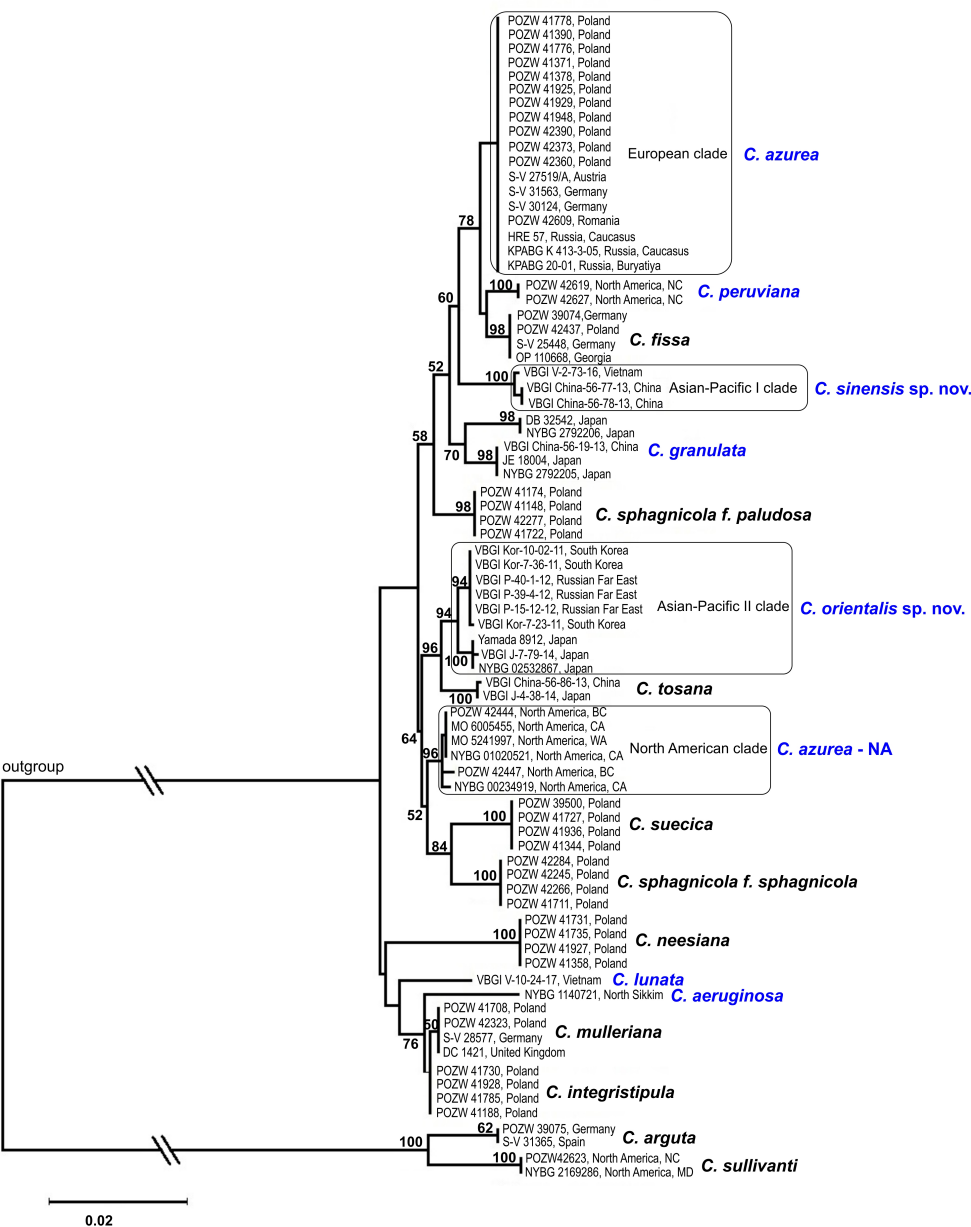
Maximum likelihood consensus tree of the studied *Calypogeia* species based on a combined chloroplast dataset. The analysis included all accessions, including those with incomplete sequences. Bootstrap values are given above the branches. Species with blue oil bodies are marked in blue.

A multigene analysis revealed high genetic diversity in plants classified as *C*. *azurea* based on the presence of blue oil bodies and morphological features. The phylogenetic analyses (NJ, MP and ML) of the combined plastid loci demonstrated that present *C*. *azurea* is a paraphyletic taxon with other species nested in *C*. *azurea* accessions. Samples of *C*. *azurea* were clustered in four different clades. The remaining species with blue oil bodies were distributed in two clades with *C*. *peruviana* and *C*. *granulata* in the first clade, and *C*. *lunata* and *C*. *aeruginosa* in the second clade. However, the second clade was weakly supported due to the presence of accessions with incomplete sequences (Fig 2 and S1 Fig).

### Differences in DNA sequences of *Calypogeia azurea*

**Fig 3:**
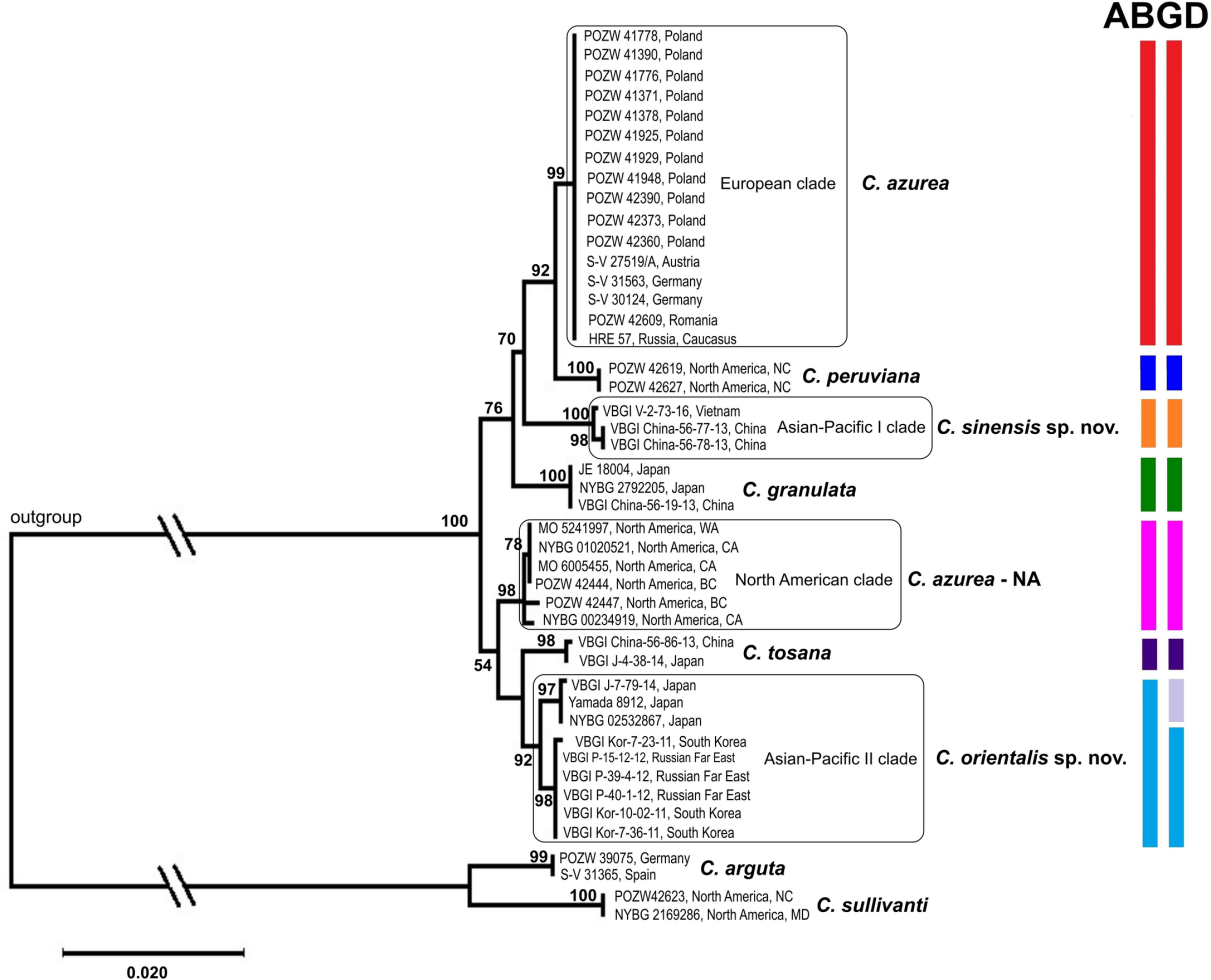
Maximum likelihood consensus tree of the *Calypogeia azurea* complex based on combined chloroplast dataset. The results of the ABGD analysis are represented by colored stripes on the right side of the diagram. The first bar represents the initial partition, and the second bar represents the recursive partition. Only the accessions containing the sequences of all loci were included in the analysis. The related taxa (*C*. *peruviana*, *C*. *granulata*, *C*. *tosana*) were used for comparison. *Calypogeia arguta* and *C*. *sullivantii* were used as an outgroup. Bootstrap values are given above the branches.

**Fig 4:**
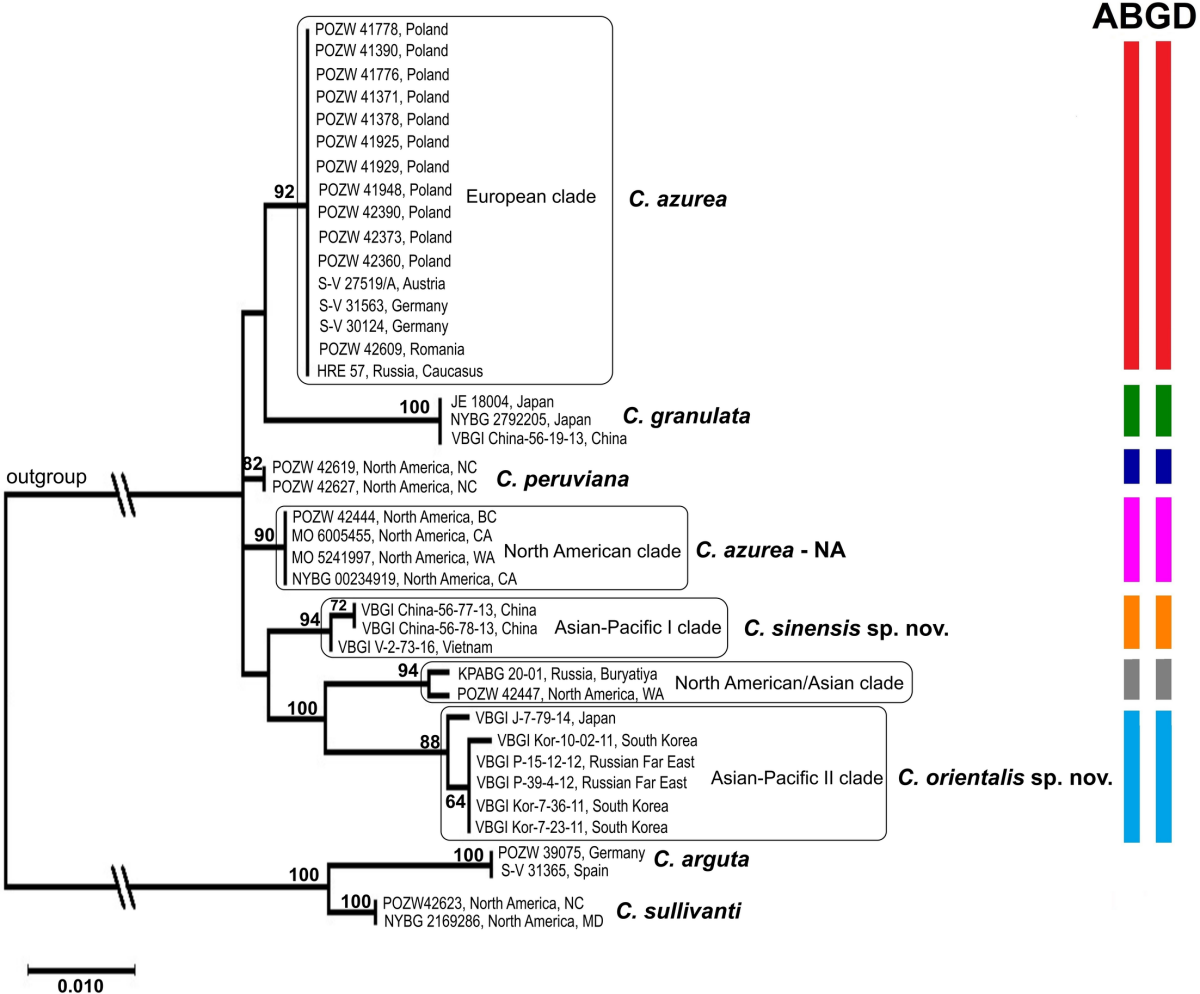
Maximum likelihood consensus tree of the *Calypogeia azurea* complex based on nuclear ITS2 sequences. The results of the ABGD analysis represented by colored stripes on the right side of the diagram. The first bar of the diagram represents the initial partition, and the second bar represents the recursive partition. Only the accessions containing the sequences of all loci were included in the analysis. The related taxa (*C*. *peruviana*, *C*. *granulata*, *C*. *tosana*) were used for comparison. *Calypogeia arguta* and *C*. *sullivantii* were used as an outgroup. Bootstrap values are given above the branches.

Subsequent analyses were limited to the *C*. *azurea* complex and other species with blue oil bodies whose accessions had complete data for all studied DNA regions. Separate analyses of chloroplast and nuclear data revealed several conflicting nodes which were only weakly supported by bootstrap values (Figs 3 and 4). The incongruence between combined plastid and nuclear data sets was confirmed by the ILD test (p = 0.001). The ILD test detected conflict between the nuclear ITS2 region and each of the four plastid regions. The relevant data were not combined, and the analyses were performed separately for chloroplast and nuclear loci.

Two independent main evolutionary lineages were formed based on the combined plastid loci (Fig 3). The first main lineage comprises the European clade of *C*. *azurea*, a sister to *C*. *peruviana*, Asian-Pacific I clade of *C*. *azurea* (described as *C*. *sinensis*) and *C*. *granulata*. The second main lineage includes the North American clade of *C*. *azurea* and Asian-Pacific II clade of *C*. *azurea* (described as *C*. *orientalis*), which is a sister to *C*. *tosana*, with 88–100% bootstrap support. In addition, *C*. *orientalis* was divided into two clusters correlated with the geographical region. The first cluster included plants from the Russian Far East and Korea, and the second cluster was composed of plants from Japan (Fig 3). The analysis of the ITS2 region revealed the same four groups of *C*. *azurea*, which, however, were bounded by different mutual relationships. The European *C*. *azurea* was resolved as a sister of *C*. *granulata*; *C*. *sinensis* formed a close relationship with *C*. *orientalis*, whereas the relationship between the North American clade of *C*. *azurea* and *C*. *peruviana* remained unresolved. The main differences between the topology of the chloroplast and the nuclear tree was the position of *C*. *azurea* KPABG 20–01 and POZW 42447 (Fig 4). These specimens belonged to the European clade and the North American clade of *C*. *azurea*, respectively, in the plastid tree, but they formed a separate clade in the nuclear tree.

**Fig 5:**
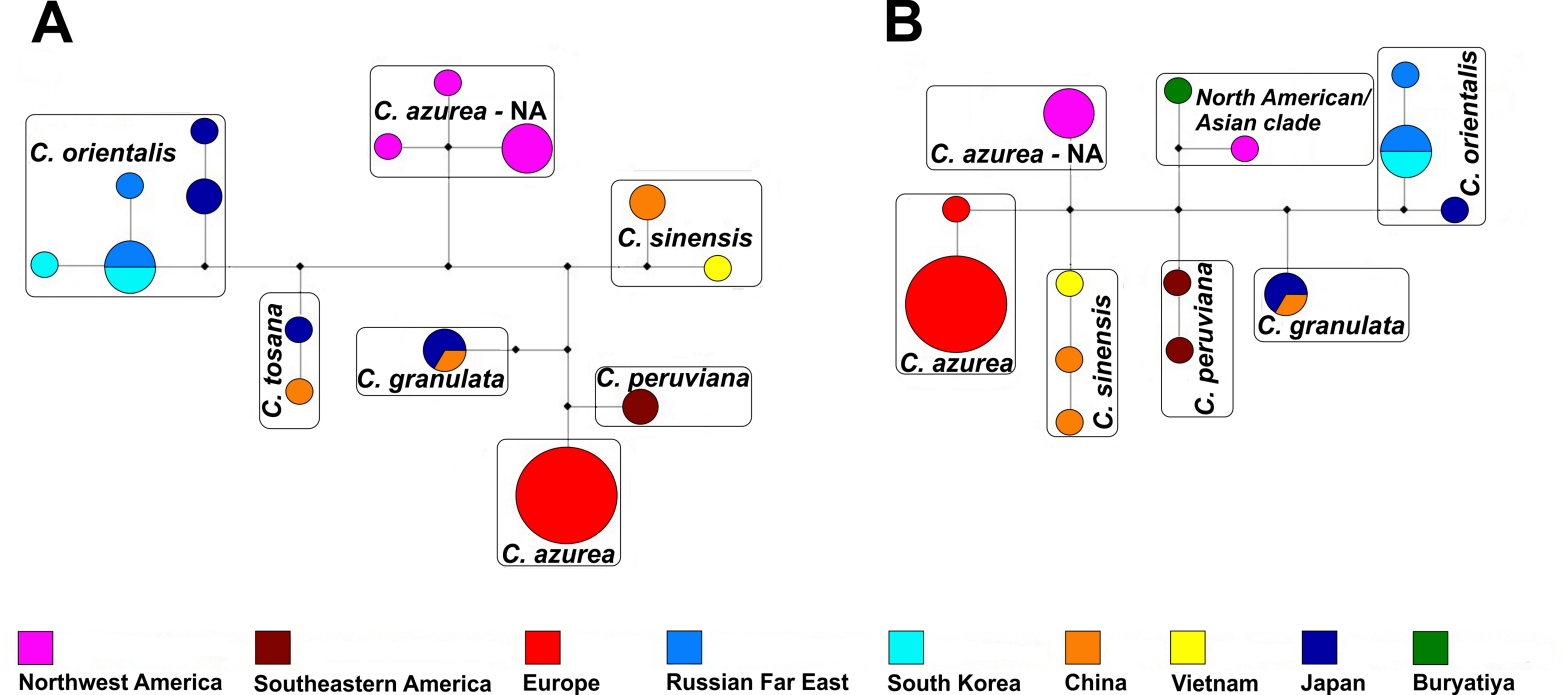
**A haplotype network of the *Calypogeia azurea* complex and the related taxa** based on combined plastid (A) and ITS2 (B) data. Colored circles represent the geographic origin of the haplotypes. Diameters denote the number of specimens carrying a particular haplotype, where the smallest circle represents a single individual, and the largest circle represents 16 individuals. Black squares represent median vectors.

Each of the four *C*. *azurea* evolutionary lineages had specific mutations that distinguished them from each other and from the remaining species with blue oil bodies (S3 Table). In the group of the analyzed *C*. *azurea* samples, 11 haplotypes were identified in both the chloroplast and the nuclear genome (Fig 5). The haplotypes were divided into four separate units corresponding to the clades identified within *C*. *azurea* in the phylogenetic trees (Figs 3 and 4). All samples from Europe (Poland, Germany, Romania, Austria), and Russia (Caucasus and South Siberia) had the same haplotype of the chloroplast DNA genome. However, only *trnL* was compared in Russian samples (based on GenBank sequences). Three haplotypes, including two from Poland and one from Russia, were detected in nuclear ITS2. Three haplotypes that differed in single substitutions were identified in the North American clade of *C*. *azurea*. Two and five haplotypes were found in *C*. *sinensis* and *C*. *orientalis*, respectively.

The Automatic Barcode Gap Discovery (ABGD) analysis was performed to split the examined specimens of *C*. *azurea* into candidate species based on pairwise distances by detecting differences between intraspecific and interspecific variation (i.e. barcoding gap) without an *a priori* species hypothesis. In the ABGD analysis, data were first divided into groups as the initial partition based on a statistically inferred barcode gap, and the same procedure was then applied to the groups obtained in the first step to form a recursive partition. The ABGD analysis based on the K2P distance model identified four and five units within the examined samples of *C*. *azurea* as the initial partition in the combined plastid loci and nuclear ITS2, respectively. This fifth unit in ITS2 comprised one sample from North America (POZW 42447) and one sample from Russia (South Siberia, KPABG 20–01). The units identified in the ABGD corresponded to the groups identified in the phylogenetic analysis (Figs 3 and 4). All examined samples were assigned to the same units as in the phylogenetic analysis. The recursive partitions based on the combined plastid loci produced 5 ABGD units because *C*. *orientalis* was split into two groups. In an analysis of individual loci, the examined samples of *C*. *azurea* were divided into 3–5 units, depending on the locus (S2 Fig). In the examined DNA regions, *C*. *granulata*, *C*. *peruviana*, *C*. *lunata*, *C*. *aeruginosa* and *C*. *tosana* formed separate units. Ultimately, four units were identified as the most reliable in the group of the analyzed *C*. *azurea* samples, and they corresponded to monophyletic groups in the phylogenetic tree.

The genetic divergence of combined plastid loci between the four lineages of *C*. *azurea* identified in the phylogenetic analysis and the ABGD analysis according to the K2P model ranged from 1.03% to 2.17%. Genetic divergence was the lowest (1.03%) between North American *C*. *azurea* and *C*. *oreintalis* lineages. The highest genetic distances (2.17%) was noted between *C*. *orientalis* and *C*. *sinensis*. All lineages identified within *C*. *azurea* differed from the remaining species, including *C*. *peruviana*, *C*. *granulata* (blue oil bodies) and *C*. *tosana*, and the genetic distances between *C*. *azurea* lineages and the above species ranged from 0.73% to 2.23% (Table 2). The lowest genetic distances were noted between the European *C*. *azurea* and *C*. *peruviana* (0.73%) and between *C*. *orientalis* and *C*. *tosana* (0.95%). The lineages of *C*. *azurea* also differed from *C*. *lunata* (3.85–5.07%) and *C*. *aeruginosa* (2.42–3.48%); however, only the *trnL* region was successfully sequenced in the above species. In the nuclear region, genetic differences between *C*. *azurea* lineages ranged from 1.00% to 2.48%, and were lowest between the European and North American lineages and highest between North American lineage of *C*. *azurea* and *C*. *orientalis* (Table 2).

**Table 2:** Average genetic differences (%) between the units identified in the *C*. *azurea* complex and the related taxa, based on the K2P combined plastid loci (below diagonal) and nuclear ITS2 (above diagonal).

	*C*. *azurea* European clade	*C*. *azurea* North American clade	*C*. *sinensis*	*C*. *orientalis*	*C*. *peruviana*	*C*. *granulata*	*C*. *azurea* North American/Asian clade	*C*. *tosana*
*C*. *azurea* European clade		1.00	1.54	2.28	0.80	2.02	2.47	-
*C*. *azurea* North American clade	1.55		1.34	2.48	0.60	2.22	2.16	-
*C*. *sinensis*	1.44	1.87		2.41	1.14	2.55	2.20	-
*C*. *orientalis*	1.89	1.03	2.17		2.28	2.69	2.24	-
*C*. *peruviana*	0.73	1.83	1.69	1.94		2.02	2.06	-
*C*. *granulata*	1.29	1.55	1.44	1.77	1.46		3.72	-
*C*. *azurea* North American/Asian	-	-	-	-	-	-		-
*C*. *tosana*	1.94	1.10	2.23	0.95	2.11	1.94	-	

### Morphological differences

**Fig 6:**
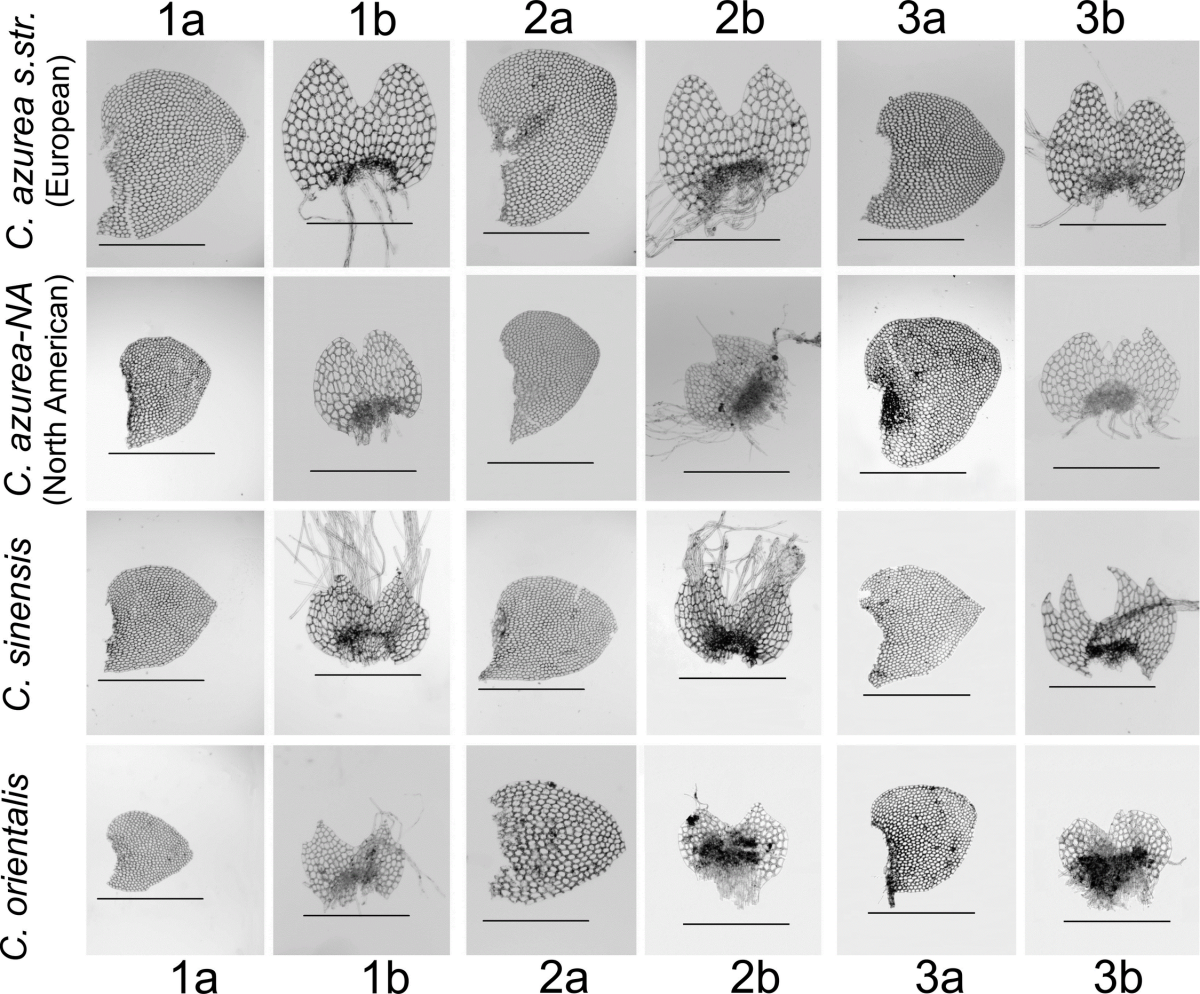
**Microscopic images of the leaves and underleaves of the *Calypogeia azurea* complex:** *C*. *azurea* s. str. (European clade, 1a, 1b –POZW 41390, 2a, 2b –POZW 41746, 3a, 3b –HRE 57); *C*. *azurea*—NA (North American clade, 1a, 1b –MO 6005455, 2a, 2b –MO5241997, 3a, 3b POZW 42444); *C*. *sinensis* (1a, 1b –VBGI China-56-78-13, 2a, 2b –VBGI China 56-77-13, 3a, 3b –VBGI V-2-73-16) and *C*. *orientalis* (1a, 1b –VBGI P-40-1-12, 2a, 2b –VBGI P-39-4-12, 3a, 3b –VBGI J-7-79-14). The samples are numbered in accordance with S1 Table. Bar = 1000 μm for leaves, bar = 500 μm for underleaves.

The groups identified within *C*. *azurea* differed in size and shape of leaves and in the shape of the lateral margin of the underleaf (Table 3, Fig 6, S4 Table, S1 Appendix). All live plants had deep blue oil bodies. The oil bodies of European and North American plants are usually segmented, composed of several 2–4 (up to 9) large and distinct globules, rarely not segmented, 4–7 × 6–12 μm, 2–7 per cell, which are present in all underleaf cells, but are absent in some leaf cells (Fig 1A–1C). The oil bodies in *C*. *azurea* were similar to those in *C*. *peruviana* (Fig 1J). The oil bodies in *C*. *orientalis* were coarsely botryoidal and somewhat similar to those observed in European and North American specimens (Fig 1D and 1E). However, the oil bodies of *C*. *sinensis* plants from the Chinese province of Guizhou (China 56-77-13 and China-56-78-13), referred to as *C*. *azurea* by Bakalin *et al*. [63], were quite different from those observed in European, North American and *C*. *orientalis* plants. They were composed of numerous small globules and may be called as coarsely granular (Fig 1F and 1G). Interestingly, the dry specimens from North America and China were similar in morphology, but they differed strikingly in the characteristics of oil bodies. A comparison of the species previously referred to as *Calypogeia* sp. (China-56-19-13; cf. Bakalin *et al*. [63]) also delivered fascinating results. Based on the results of the DNA sequence analysis, this specimen was included in the *C*. *granulata* clade, and it was characterized by gray oil bodies (which is sometimes observed in the species). The oil bodies in the latter species were finely (not coarsely) granular (Fig 1I).

**Table 3:** Morphological differences between the units detected within the *C*. *azurea* complex and the related taxa.

Feature	*C*. *azurea* s. str.	*C*. *azurea–*NA	*C*. *orientalis*	*C*. *sinensis*	*C*. *granulata*
Underleaf shape	Lobe apices subrounded, not incised, undivided area with 3–6 cells high, lateral side smooth	Lobe apices subrounded, not incised, undivided area with 2–4 cells high, lateral side smooth	Lobe apices merely acute, lateral sides usually have additional teeth, or lobe apex incised, undivided area with 1–3 cells high	Deeply divided into two triangular lobes, with lateral side rounded (rarely), crenulate or with distinct, although obtuse teeth to bisbifid, undivided area with 2-3(-4) cell high	Obligately bisbifid
Underleaf size	Well-developed underleaves, 490–950 μm wide × 400–790 μm long	Well-developed underleaves, 300–770 μm wide × 250–690 μm long	Well-developed underleaves, 400–800 μm wide × 200–300 μm long	Well-developed underleaves, 300–550 μm wide × 400–1000 μm long	Well-developed, underleaves 200–600 μm wide × 200–400 μm long
Oil bodies	Oil bodies deep to pale blue, present in all cells of underleaf cells, but absent from some leaf cells, usually composed of 2–4 (-9) large and distinct globules, (2-)3-7 per cell, 4-7x6-12 μm	Oil bodies deep to pale blue, present in all cells of underleaf cells, but absent from some leaf cells, usually composed of 2–6 large and distinct globules or rarely spherical 2–3 per cell, 4-7x6-12 μm	Oil bodies botryoidal in the middles, dark blue, 2(-3) per cell, ellipsoidal, irregularly oblong to shortly fusiform, 4-11x3-5 μm	Composed of small globules (coarsely granular), deep blue to blue-brown, 3–6 per cell	Deep blue to brown-blue and brownish, finely granular
Dominant ecology	Moist humus and soils	Decaying wood, organic debris	Decaying wood	Cliffs to decaying wood and tree trunks	Moist cliffs and soils

### Taxonomic conclusions and description of new species

The DNA sequence analysis broadened the concept of the *Calypogeia azurea* complex. Individual clades of *C*. *azurea* are more closely related to other recognized species than to each other (Figs 2–4). The observed level of genetic divergence and the morphological, ecological and geographical differences indicate that these clades can be regarded as previously unrecognized species. The two evolutionary lineages discovered in Pacific Asia differed morphologically and can be described as new *Calypogeia* species with blue oil bodies. The plants in the Asia-Pacific I clade were described as *C*. *sinensis*, whereas the plants in the Asia-Pacific II clade were described as *C*. *orientalis*. Due to the lack of diagnostic morphological features, the European and North American lineages, must be temporarily treated as cryptic species of *C*. *azurea*. The European plants were named as *C*. *azurea* s. str., whereas North American plants as *C*. *azurea* species NA.

#### *Calypogeia azurea* Stotler & Crotz

The plants belonging to the European lineage were morphologically similar to the holotype of *C*. *azurea* Stotler & Crotz described by Stotler & Crotz [64] based on a specimen of K. Müller from KR herbarium, i.e. *Calypogeia trichomanis* Corda, Ölkpr. blau! (Auf Erde … am Feldberg, ca 1000 m, 1.5.1946, K. Müller s.n. (KR), Fig 2G–2I) [64]. The examined plants of *C*. *azurea* from Europe had cordate to broadly cordate leaves, with usually greater width than length, with a broadly rounded or subacute apex (Fig 6). Underleaves were wider than the stem, suborbicular to ovate, divided by an acute sinus, with lateral margins evenly rounded or rarely with vague angulations (Fig 6). The plants of North American lineage were morphologically similar to the European plants, but the European specimens were larger than North American plants, and the average width of leafy shoots was determined at 2000 and 1678 μm, respectively. The underleaves of European and in North American specimens were wider than the stem at 680 and 580 μm, respectively, on average (Fig 6). *Calypogeia azurea* was monoicous, mainly autoicous, and often fertile. The studied plants grew mainly on moist humus, peaty, clay or sandy soil, organic debris, rocks with a layer of humus (in Europe) and on decayed logs (in North America). The analyzed specimens were distributed throughout Europe (Poland, Austria, Germany, Romania, European and South Siberian Russia) and North America (Canada—British Columbia, USA—California and Washington). *Calypogeia azurea* also occurs in the eastern North America, but our samples did not include specimens from this region.

#### *Calypogeia sinensis* Bakalin & Buczkowska, sp. nov. (Fig 7)

**Description:** Plants prostrate, lax and soft, in loose pure mats or with other hepatics or mosses, yellow-brown in a herbarium, blue-green to pale green when alive, 20–50 mm long and 2.0–3.2 mm wide. Rhizoids brownish, erect to obliquely spreading, united in unclear fascicles, rarely separated. Stem rarely to regularly branched, with ventral branches, branch base partly covered by the underleaf, 1(-2) per underleaf. The stem cross-section transversely elliptic, 180–300 × 250–500 μm, with the external wall slightly thickened, cortex not prominent to virtually absent, outer cells on the dorsal side 25–36 μm along the margin (the cells appeared elongated along the margin in cross-section view, and were transversely elliptic in outline), with moderate to small in size trigones and slightly thickened walls (thin walls were rare). Inner cells subisodiametric, 20–50 μm in diameter, thin-walled, with small trigones, ventral epidermal cells similar to dorsal epidermal cells, but cells more or less isodiametric in the cross-section, with thickened and brown-colored walls. Leaves contiguous to loosely imbricate, slightly convex, loosely undulate along the margins, with the apical part turned to the dorsal side, obliquely to subhorizontally inserted, dorsally insertion line arcuate, not decurrent, ovate to widely ovate, apex acute to shortly bidentate, 1.1–1.5 × 1.0–1.5 mm. Underleaves obliquely spreading, transversely elliptic, 300–550 × 400–1000 μm, deeply divided into two triangular lobes, with the lateral side rounded (rarely), crenulate or with distinct but obtuse teeth (then underleaves bisbifid), undivided area between the bottom of the sinus and the initial rhizoid area with 2-3(-4) cell high; lobe apex commonly with obovate slime papilla. Cells in the midleaf shortly oblong to subisodiametric 5-6-gonal, 35–55 × 30–43 μm, trigones absent or vestigial; cells along the leaf margin elongated along margin or isodiametric, 30–50 μm long, cuticle smooth throughout the leaf, oil bodies coarsely granular, deep blue to blue-brown, 3–6 per cell. Asexual reproduction by gemmae at the apices of attenuate tips, spherical unicellular to ellipsoidal bicellular, 20–23 μm in diameter or 20–33 × 20–23 μm, colorless to greenish. Good for holotype: China-56-77-13.

**Fig 7:**
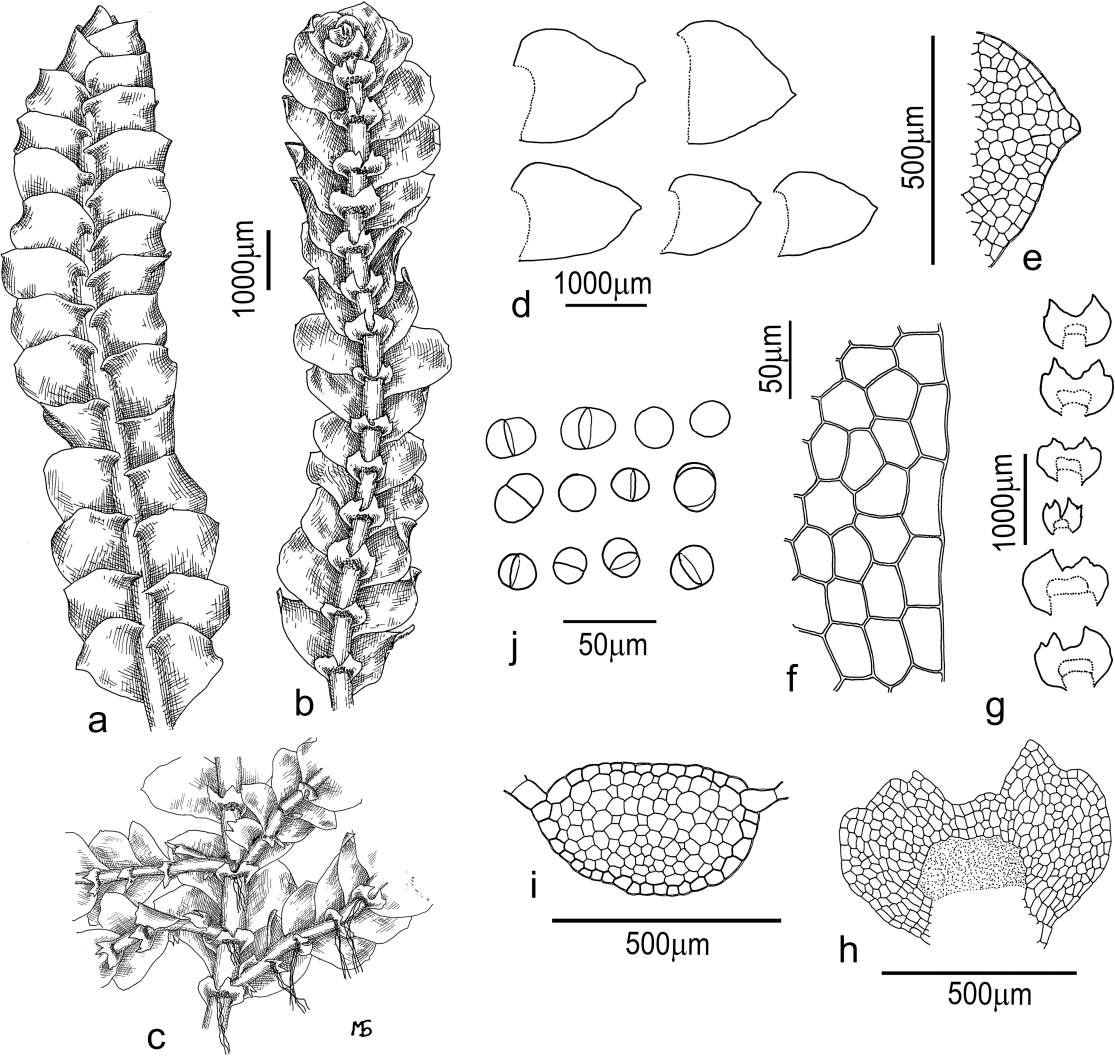
***Calypogeia sinensis* Bakalin & Buczkowska**: (a) plant segment, dorsal view; (b-c) plant segment, ventral view; (d) lateral leaves; (e) leaf apex; (f) leaf margin (40×); (g-h) underleaves; (i) stem cross sections (10×); (j) oil bodies (40×). Sample VBGI China-56-77-13.

**Holotype:** China. Guizhou Province. Duyun Municipality (26°22’23”N 107°21’21”E), 1300 m alt., VB 22.XI.2013 (China-56-77-13, VBGI, duplicate in POZW).

**Paratypes:** China. Guizhou Province. Duyun Municipality (26°22’23”N 107°21’21”E), 1300 m alt., VB 22.XI.2013 (China-56-78-13, China-56-96a-13, VBGI, duplicate in POZW).

Vietnam. Lao Cai Province. SaPa, Phan Xi Pan National Park (22°20'18"N 103°46'39"E), 1900–2100 m alt. VB 16.III.2016. (V-2-73-16; VBGI, duplicate in POZW).

**DNA sequences:** Samples China-56-78-13, China-56-77-13 and V-2-73-16 were used to sequence 4 chloroplast regions (*rbcL*, *trnH-psbA*, *trnL* and *trnG*) and one nuclear (ITS2) region. The sequences were deposited in GenBank. Accession numbers are given in S1 Table.

**Differentiation:** In the morphological terms, the most close relative to *C*. *sinensis* is *C*. *granulata* that moreover has sympatric area with the former species in the Chinese province of Guizhou. The typical *C*. *granulata* (including the isotype G00114896!) differed considerably from *C*. *sinensis*. The underleaves of *C*. *granulata* were narrow (1.5–2.0 times wider than the stem) and obligately bisbifid, whereas in *C*. *sinensis*, the underleaves were 2.5–3.5 times wider than the stem, commonly bifid or with smaller additional teeth on each side. Oil bodies were coarsely granular (almost botryoidal) in *C*. *sinensis* and finely granular in *C*. *granulata* (S1 Appendix).

**Ecology:** Very little is known about the ecology of *C*. *sinensis*. According to the literature, the taxon prefers wet open cliffs and partly shaded cliff caves in the waterfall spray zone, as well as mesic tree trunk bases and decaying wood in broadleaved, evergreen, subtropical to southern subtropical mountane forests.

**Distribution:** There are only two known localities of the taxon. The first is the subtropical montane forest near Xiniu Waterfall in Chinese province of Guizhou, where the species was collected at the estimated altitude of 1300 m. The second locality is a subtropical montane forest in southern North Vietnam, where the species was collected at the estimated altitude of 2000 m. The taxon probably belongs to the ‘meta-Himalayan’ group of the species [65] that could colonize other parts of the southern-eastern spur of the Tibetan Upland (e.g. Yunnan Province) as well as North Vietnam.

#### *Calypogeia orientalis* Buczkowska & Bakalin, sp. nov. (Fig 8)

**Description:** Plants soft, green to blue-green (blue coloration was particularly visible near the shoot tips) when alive, yellow-green, golden brown to brown-green in a herbarium. Plants grew in dense to loose pure patches or as admixture to mosses and some hepatics. They were 5–30 mm long and 1.5–2.5 mm wide, creeping to loosely ascending near apices. Rhizoids pale brown, obliquely upward spreading (in the direction of the shoot apex, not downward) or erect spreading, mostly united into unclear fascicles. Stems rarely and irregularly ventrally branched; with a transversely elliptic cross-section, 180–220 × 250–300 μm. The cortex not prominent, with epidermal cells on the dorsal side, 15–38 μm in diameter, to the middle, 17–30 μm in diameter; 5-6-gonal, epidermal cells on the ventral side 12–25 μm in diameter; across the entire cross-section, cells thin-walled, and dorsal (rarely ventral) epidermal cells and adjacent cell rows sometimes loosely thickened and brownish in color; trigones small to virtually absent, more pronounced on the dorsal side. Leaves imbricate, obliquely inserted, dorsally insertion line arcuate, not or barely decurrent, leaves nearly plane to slightly concave and loosely canaliculate, with the apex usually slightly turned to the ventral side; widely ovate to slightly obliquely ovate when flattened on the slide -, 0.7–1.2 × 0.7–1.3 mm; the apex acute, sometimes shortly bilobed, rarely rounded. Underleaves loosely appressed to the stem or obliquely spreading, decurrent along 1/3-1/2 of stem width, obliquely elliptic in outline, 200–300 × 400–800 μm, deeply divided into two triangular lobes with obovate slime papillae in the apex, the lateral side entire to loosely crenulate or with an additional obtuse tooth on each side, rarely bisbifid, the undivided portion between the bottom of the sinus and rhizoid initial area with 2-3(-5) cells high. Mid-leaf cells 5-6(-7)-gonal, subisodiametric to shortly oblong, 37–63 × 25–45 μm, thin-walled, trigones vestigial; cells along the leaf margin oblong, 30–48 × 20–30 μm, trigones adjacent to external side, moderate in size and concave; the cuticle smooth across the entire leaf; oil bodies botryoidal, dark blue, 2(-3) per cell, ellipsoidal, irregularly oblong to shortly fusiform, 4–11 × 3–5 μm. Dioicous: only two young archegonia on short ventral branches were observed in the holotype.

**Fig 8:**
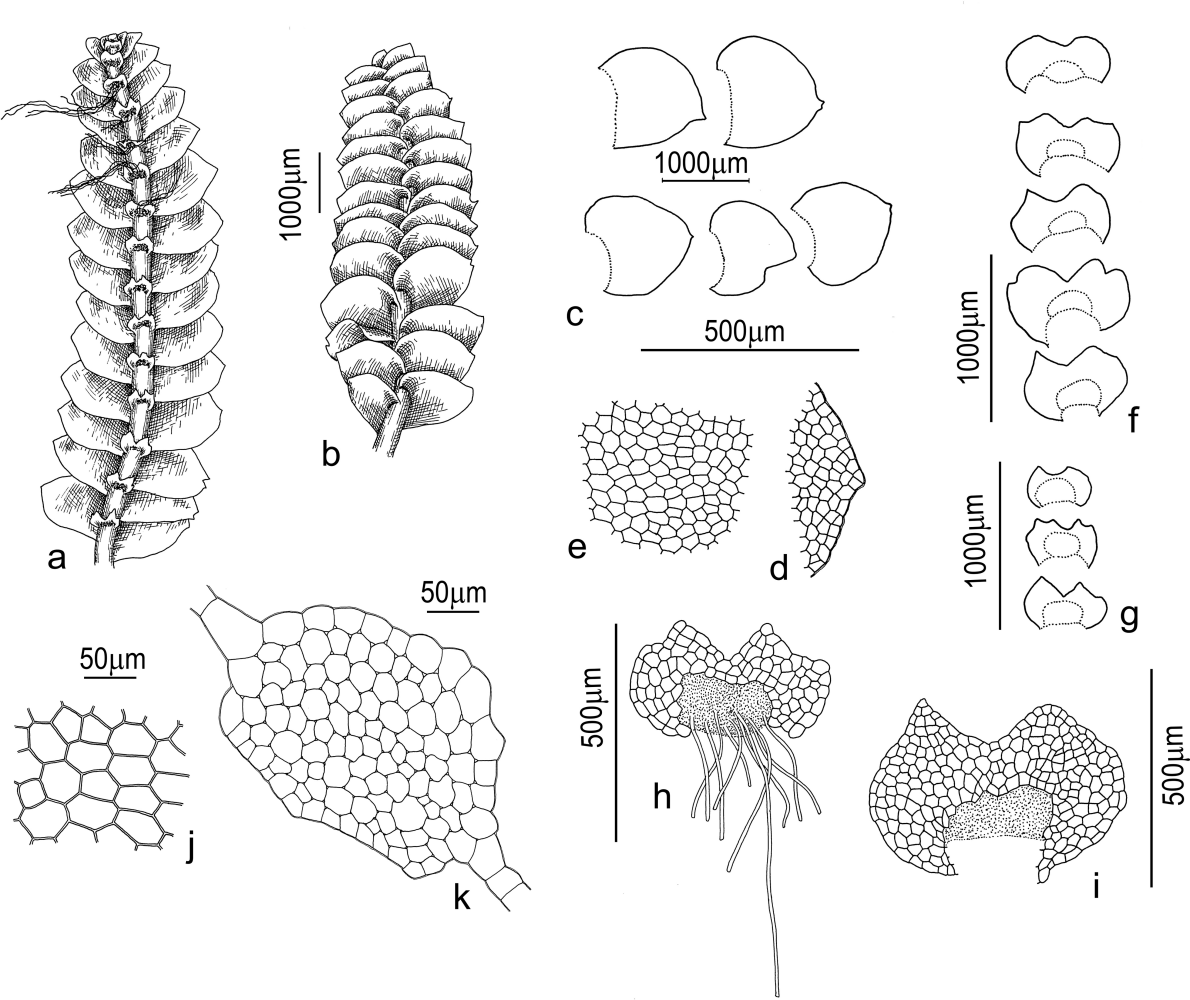
***Calypogeia orientalis* Buczkowska & Bakalin**: (a) plant segment, dorsal view; (b) plant segment, ventral view; (c) lateral leaves; (d) leaf apex (10×); (e) median leaf cells (10×); (f-i) underleaves; (j-k) stem cross sections (40×). Samples: VBGI P-39-4-12 (parts a, c, d, f, h, i, j, k), VBGI Kor-7-36-11 (parts b, e, g).

**Holotype:** Russia. The Russian Far East. Primorsky Territory. Shkotovsky District (43°07’08”N 132°45’45”E), 450 m alt., VB 18.IX.2012 (P-39-4-12, VBGI, duplicate in POZW)

**Paratypes:** Japan. Fukuoka Prefecture, Tagama-gun (33°28’20”N130°54’07”E), 800–900 m alt., VB 17.III.2014 (J-7-79-14, VBGI, duplicate in POZW).

Republic of Korea. KangWon Province (38°07’40”N 128°26’55”E), 1250 m alt., VB 11.V.2011 (Kor-7-23-11, Kor-7-36-11, VBGI, duplicate in POZW), (38°07’33”N 128°27’22”E), 1550 m alt., VB 12.V.2011 (Kor-10-02-11, VBGI, duplicate in POZW); KyongNam Province (35°19’42”N 127°43’07”E), 1300–1600 m alt., VB 07.V.2015 (Kor-28-1-15, Kor-28-3-15, VBGI).

Russia. The Russian Far East. Primorsky Territory. Khasansky district (43°07’00”N 131°29’15”E), 140 m alt., VB 10.VII.2015 (P-39-4-15, P-39-3-15, VBGI), (43°05’18”N 131°31’19”E), 330 m alt., VB 11.VII.2015 (P-41-8-15, P-41-10-15, VBGI); Shkotovsky District (43°06’57”N 132°47’26”E), 490 m alt., VB & K.G. Klimova 15.X.2016 (Prim-16-10-16, VBGI), (43°07’10”N 132°47’31”E), 550 m alt., VB (P-15-12-12, VBGI, duplicate in POZW), (43°07’08”N 132°45’45”E), 400 m alt., VB (P-40-1-12, VBGI, duplicate in POZW). Sakhalinskaya Province. Iturup island (45°29’46”N 148°49’31”E), 750 m alt., VB 16.IX.2015 (K-77-20-15, VBGI), (45°21’45”N 148°37’13”E), 23 m alt., VB 10.IX.2015 (K-70-3-15, VBGI).

**DNA sequences:** Samples P-15-12-12, P-39-4-12, Kor-7-23-11, Kor-10-02-11, Kor-7-36-11, J-7-79-14 were used to sequence 4 chloroplast regions (*rbcL*, *trnH-psbA*, *trnL* and *trnG*) and one nuclear region (ITS2). The sequences were deposited in GenBank. Accession numbers are given in S1 Table.

**Differentiation:** *Calypogeia orientalis* is closely morphologically related to *C*. *azurea*, but the latter species differed from *C*. *orientalis* in the following features: 1) generally wider plants, where well-developed plants had a width of 2.2–2.8 mm wide (smaller if depauperate), 2) more lax-textured plants, with loosely undulate leaves, 3) larger and wider leaves reaching 1.5 × 1.8–2.0 μm in well-developed shoots, 4) larger midleaf cells that commonly reach 52–80 × 45–55 μm, 5) subrounded, not bisbifid underleaf lobe apices, undivided area with 5–6 cells high, size of well-developed underleaves 0.6–0.7 × 0.9–1.0 mm, 6) epidermal cells in the stem cross-section were larger, around 30–45 μm in diameter, inner cells 25–48 μm in diameter, 7) leaves were contiguous, not or very loosely imbricate, 8) leaves subhorizontally (not obliquely) inserted, 9) *C*. *azurea* thrives on fine soils along streams or on humificated soil, whereas *C*. *orientalis* prefers decaying wood and rarely occurs on soil. The relationships between *C*. *orientalis* and *C*. *tosana* poses a more intricate question. The two species are the closest genetic relatives despite considerable differences in oil bodies. The oil bodies of *C*. *tosana* were colorless, whereas the oil bodies of *C*. *orientalis* had a distinct deep blue color. However, oil bodies were absent from dry herbarium specimens, which makes the discussed species difficult to distinguished. The unclear concept of *C*. *tosana* further complicates matters. This species is regarded as quite polymorphic, and it has two modifications–with smooth or faintly verruculose cuticle. The analyzed specimens were studied in living conditions and they were characterized by colorless oil bodies (Fig 1l) and smooth cuticle (which was also observed in holotype G00047274/26013!). Therefore, in addition to the color of the oil bodies, as the main distinguishing feature, these two taxa also differed in almost constantly bisbifid underleaves and shortly bilobed leaves in *C*. *tosana* (rarely noted in *C*. *orientalis*). Thus, the morphological delimitation of these species is difficult, and it could be impossible in the absence of live material.

**Ecology:** *Calypogeia orientalis* is an acidophilic mesophyte. It is found mainly on decaying wood in old-growth hemiboreal mixed (coniferous-broadleaved) forests and in corresponding mountain belts. The species spreads widely to oroboreal (corresponding to southern taiga) *Abies*-*Picea* forests in the hemiboreal zone. It is less frequently noted in cool-temperate forests, where it is mostly confined to middle mountain belts in the south. The species occurs rarely in upper elevation belts (tundroids, but never true mountain tundra) in the hemiboreal zone, but above the timberline in wet landscapes, commonly surrounded by *Pinus pumila* (LPall.). In southern Kurils, the species occurs only in Regel thickets. *Calypogeia orientalis* occasionally colonizes swamps. It is rarely found on fine soils along streams and or on partly shaded moist cliffs in the orotemperate mountain belt of the Korean Peninsula. The species forms pure marts or grows with mosses and other hepatics such as *Liochlaena subulata* (A.Evans) Schljakov. In swampy habitats, the species sometime overgrows *Sphagnum* and then associated with *Scapania paludicola* Loeske et Müll.Frib. In wet fine soils, the species may occurs together with *Cephalozia otaruensis* Steph.

**Distribution:** The northernmost localities of the species are known from Southern Kurils, but the species rarely occurred beyond 48°N. The species seems to be more common in the latitude range of 43 to 35°N. The southernmost (confirmed by DNA) locality was determined at 33°N in Hiko-san Mountain, Fukuoka Prefecture, Japan. Site elevation began at near sea level in northern extremes (where the species has been found in localities as high as 750 m), and increased gradually with a decrease in latitude. The highest localities (800–1550 m) were found in the central part of South Korea. According to available data, *C*. *orientalis* has not been confirmed in North Korea or north-east China, where it is expected to be found in the future.

## Discussion

### Molecular evidence for the presence of unrecognized species within *C*. *azurea*

Live specimens of *C*. *azurea* are easy to identify based on the presence of blue oil bodies. Another characteristic feature are turquoise-blue shoot apices, which make *C*. *azurea* easy to recognize at a glance in the field. Due to the ease of identification, *C*. *azurea* was not considered as a critical species that required special attention. However, due to high morphological variation within *C*. *azurea*, herbarium specimens lacking oil bodies were frequently confused with other species, e.g. *C*. *fissa*, *C*. *muelleriana* or *C*. *tosana* [1, 17, 66].

The results of our study revealed high genetic diversity of *C*. *azurea* in the present taxonomic status. The phylogenetic trees constructed based on both combined plastid loci (*rbcL*, *trnL*, *trnG*, *trnH-psbA*) and the nuclear ITS2 region revealed that other *Calypogeia* species are nested within the examined *C*. *azurea* accessions (Figs 2–4). The samples that were initially identified as *C*. *azurea* were split into four well-supported clades (BS 88–100%) that correlate with geographic distribution. European, North American, and two (I and II) Asian-Pacific evolutionary lineages were distinguished. The same four lineages were detected in the ABGD analysis with the use of the software developed by Puillandre *et al*. [58], which groups examined samples into several ABGD units (hypothetical species) with the use of pairwise differences. Identical four groups were obtained in network analysis. The genetic divergence between the four lineages of *C*. *azurea* ranged from 1.03% to 2.17%. The lowest divergence (1.03%) was noted between the North American lineage (*C*. *azurea*—NA) and *C*. *orientalis*. Although the divergence between the detected groups did not exceed 2% or 3%, which is often proposed as a threshold in comparisons of congeneric species pairs in barcoding studies [58, 67], we think that the four evolutionary lineages of *C*. *azurea* should be considered as separate species. Their species rank is supported by the presence of other species nested between the four *C*. *azurea* lineages. Phylogenetic reconstruction suggests that blue oil bodies in *Calypogeia* appeared at least twice during its evolution (Fig 2).

### The relationship between European and North American *C*. *azurea*

The morphological features of European plants correspond to the *C*. *azurea* holotype described by Stotler & Crotz [64] based on K. Müller specimens. The holotype originated in Germany, therefore, the name *C*. *azurea* Stotler & Crotz can only be assigned to the European lineage. The North American lineage is sufficiently genetically different to be distinguished as a separate species and provided with a new name; however, the species cannot be formally described due to the lack of diagnostic morphological features (Fig 6). These plants also had a similar structure of oil bodies (Fig 1A–1C). They differed only in size and ecological preferences. Further research involving more material is needed to describe the morphological features for identifying the discussed species. For this reason, the North American species has to be temporarily named as *C*. *azurea* species NA.

The genetic distances between the two lineages of *C*. *azurea* in the examined plastid DNA regions was 1.55%. This value approximates the differences reported in other accepted *Calypogeia* species, including *C*. *azurea* and *C*. *sphagnicola* [43]. Clear genetic differences between the North American and European lineages of *C*. *azurea* were also found in isozyme markers, with limited gene flow between the populations originating from North America and Europe [45]. The existing data also indicate that the species occurred on different substrates. The DNA sequences of the above lineages of *C*. *azurea* differed from those of *C*. *peruviana*, the second North American species with blue oil bodies which, according to Schuster [1], is sometimes difficult to distinguish based on morphological features. It is worth noting that in terms of chloroplast genome sequences, *C*. *peruviana* is a sister group to the European lineage of *C*. *azurea*, and the genetic divergence between these species was lowest among all compared species pairs at 0.73% (Table 3). This relationship was not confirmed by ITS2 phylogeny due to the lack of resolution at the backbone. There is a certain degree of discordance in the relationships between the groups distinguished based on plastid and nuclear markers, and the most greatest differences were noted in the location of two *C*. *azurea* samples (KPABG 20–01 and POZW 42447) as well as in the phylogenetic relationships between both clades (European and North American) of *C*. *azurea* and *C*. *peruviana*. Sample KPABG 20–01 had the European haplotype and sample POZW 42447 had the North American haplotype of chloroplast markers, but they were located in a new clade in ITS2 phylogeny. According to Soltis & Soltis [68], topological incongruence between plastid and nuclear phylogeny can be caused by hybridization. Cytological and isozyme studies have demonstrated that European plants of *C*. *azurea* have allopolyploid origin [69–70], therefore, the obtained results could suggest that *C*. *peruviana* and the European *C*. *azurea* inherited chloroplasts from a shared parent, whereas the North American and European lineages of *C*. *azurea* most likely had different parents as donors of their chloroplast genomes. The European and North American *C*. *azurea* most probably evolved during two independent hybridization processes, which would confirm their status of separate species. However, the number of chromosomes (n = 18) was determined only in European material [69–71], and the polyploidy status of North American plants remains unknown. The existing data do not support the reconstruction of the hybridization process. Nevertheless, the observed differences in the DNA sequences of European and North American lineages indicate that they are separate species. However, due to the lack of reliable diagnostic morphological features, they are treated as the cryptic species of *C*. *azurea*.

### Differences between *C*. *azurea* and new described species

The two evolutionary lineages discovered in Pacific Asia, *C*. *orientalis* and *C*. *sinensis*, can be described as new *Calypogeia* species. Genetic divergence between these species was determined at 2.17%. In terms of DNA sequences, both new species also differed from *C*. *peruviana*, *C*. *granulata*, *C*. *lunata* and *C*. *aeruginosa*, other *Calypogeia* species with blue oil bodies [7, 9, 22], and from the compared species of *C*. *tosana* (Figs 2–4, S1 and S2 Figs). Genetic distances between the newly recognized species ranged from 0.95% (between *C*. *orientalis* and *C*. *tosana*) to 2.23% (*C*. *sinensis* and *C*. *tosana*). Further evidence supporting the species rank of *C*. *sinensis* and *C*. *orientalis* comes from morphological studies, which revealed compatibility between the genotype and the phenotype. A detailed analysis of the samples identified by barcode markers demonstrated clear morphological differences between the distinguished lineages of *C*. *azurea* that had been previously overlooked. Both new species (*C*. *sinensis* and *C*. *orientalis*) can be distinguished from one another and from the European and North American lineages of *C*. *azurea* based on the presence of diagnostic traits, both morphological and those related to oil bodies. The major diagnostic features were the shape and the size of leaves and underleaves (Figs 6–8, S4 Table). Differences in ecological preferences provided additional evidence for the separateness of the taxa. *Calypogeia orientalis* occurs mainly on decaying wood in old-growth hemiboreal mixed forests (coniferous-broadleaved) at lower altitudes in the north, and in the oro-hemiboreal mountain belt in the south. *Calypogeia sinensis* generally occupies different habitats than both *C*. *azurea* s. str. and *C*. *orientalis*. It is an element of evergreen montane subtropical forests, where it colonizes wet open cliffs and partly shaded cliff caves. Very little is known about the ecology of *C*. *sinensis*, but one of authors (VB) never observed “*C*. *azurea*” s.l. growing on decaying wood in southern China or North Vietnam. Both new species occupy different habitats than the European *C*. *azurea*, which grows mainly on moist humus, peaty, clay or sandy soil, organic debris and rocks with a layer of humus, and the North American *C*. *azurea* which colonizes decayed logs. In addition, the genetic diversity of *C*. *azurea* was also confirmed by differences in the chromosome number which was determined at n = 18 and n = 9 in European and Asian *C*. *azurea*, respectively [69, 72–76].

### Differentiation within species

The four identified lineages of *C*. *azurea* differed in the level of genetic variation. European plants did not differ in plastid DNA sequences. The samples of *C*. *azurea* originating from different parts of Europe shared one haplotype of all analyzed plastid DNA regions. The sample from the Republic of Buryatiya (KPABG 20–01) had the same haplotype as European plants, but only the sequence of one DNA region (*trnL*) was available in GenBank. The sample differed in 9 substitutions in the ITS2 sequence. Unlike European specimens, the samples from North America were characterized by 0.12% variation within the lineage, with three different haplotypes in plastid loci and two haplotypes in ITS2. Similar differences between the North American *C*. *azurea* were found in isozyme markers [45]. Intraspecific variability was also detected in the two new species, *C*. *sinensis* (0.18%), and *C*. *orientalis* (0.27%). Within *C*. *orientalis*, two sister lineages were observed in the phylogenetic tree. The first consisted of plants from Korea and the Russian Far East, and the second comprised plants from Japan. Our results confirm the observations made by [8] who found that the speciation of the genus *Calypogeia* occurred on a very regional scale. According to Inoue [8], most of the Japanese species of *Calypogeia*, including *C*. *nasuensis*, *C*. *asakawana*, were restricted to very small areas. On the other hand, most European species had much broader ranges [1, 16, 18], which could be attributed to extensive shield glaciations at the end of the Pleistocene which contributed to the homogeneity of the local flora. Our results suggest that only one haplotype of *C*. *azurea* could be distributed throughout Europe, whereas several haplotypes are distributed in the Pacific East Asia.

### Geographic distribution

To date, *C*. *azurea* was generally regarded as a species of the Holarctic range, and it was reported from various parts of the northern hemisphere, from North America throughhout Europe to the Far East [1, 16, 18, 20, 63]. Our results revealed that plants corresponding to *C*. *azurea* according to Stotler & Crotz [64] had a more restricted geographical distribution than previously thought. All the species which emerged after the splitting of *C*. *azurea* s.l. have allopathic ranges. The actual *C*. *azurea* s. str. (corresponding with type) occurs in Europe, whereas the morphologically similar cryptic species provisionally named as *C*. *azurea* species NA occurs in the Pacific Northwest. The geographical range of the newly recognized *C*. *orientalis* species is probably restricted to hemiboreal and temperate Pacific East Asia. It covers the southernmost flank of the Russian Far East, Korean Peninsula and Japanese archipelago, where insular populations are somewhat different from the continental populations. The distribution of *C*. *sinensis* is probably confined to south-eastern Tibetan Plateau in southern China and Vietnam. The existing data indicate that the four species separated from the former *C*. *azurea* s.l. had allopatric distribution. The geographic distribution of *C*. *azurea* and *C*. *orientalis* could potentially coincide in south-eastern Siberia, although the odds are relatively low. There are no reports of *C*. *azurea* in the Upper Amur region, also in available herbarium collections from this region. The distribution pattern of many Euro-Siberian taxa, including *Pinus silvestris* L., is similar, and their geographic range ends in the Upper Amur region and does not continue eastward towards the true East Asian communities. There is growing molecular evidence to suggest that the morphologically defined liverwort species are, in fact, composed of several unrecognized species with a more limited geographic distribution, including *Conocephalum conicum* [77], *Pellia epiphylla* [78], *Corsinia coriandrina* [79], *Frullania tamarisci* [29], *Porella platyphylla* [30] and species of the genera *Herbertus* [32] and *Plagiochilla* [34]. In some cases, species morphology was re-examined based on the results of recent genetic research, which led to the identification of new species, or the revision of the existing species, such as *Conocephalum conicum* and *C*. *salebrosum* [77], *Herbertus borealis* and *H*. *norenus* [32].

### Relationship between newly distinguished and other *Calypogeia* species

Our studies revealed a close relationship between *C*. *tosana* and the newly distinguished species *C*. *orientalis* and *C*. *azurea—*NA. Genetic divergence between these species in the examined DNA regions is relatively low (0.95% and 1.10%, respectively). However, both new species differ distinctly from *C*. *tosana* in the color and structure of oil bodies (colorless and granular in *C*. *tosana*, while blue and composed of distinct globules in *C*. *azurea*—NA and *C*. *orientalis*), which argues for keeping the rank of species for all three taxa. In liverworts, the structure and color of oil bodies are considered a strong and one of the most important taxonomic features used for the delimitation of species. It should be emphasized, however, that the correct delimitation of these species is possible only in the living state, so the species must be identified before drying the plants. However, our results indicate that *C*. *tosana* requires further molecular and morphological studies.

The relationship between European *C*. *azurea* and *C*. *peruviana* has not been resolved either. The species were characterized by low (0.73%) genetic divergence in the analyzed DNA regions, and the question that needs to be answered is whether these differences are sufficient to regard both taxa as separate species. The species status of *C*. *azurea* and *C*. *peruviana* was supported by differences in isozyme markers, especially the presence of fixed heterozygotes [45, 70] which point to their allopolyploid origin. In this study, *C*. *peruviana* material was very limited, which prevented an evaluation of its genetic structure. For this reason, we decided to maintain the species rank for both taxa at the current stage of research. The results of molecular analyses indicate the need for a general revision of the genus *Calypogeia*.

## Supporting information

S1 Fig **Neighbor joining (A) and maximum parsimony (B) consensus trees of the *Calypogeia azurea* complex.** The analyses were performed separately for a combined plastid loci and nuclear ITS2. Only the accessions with complete sequences of all loci were included in the analysis. The related taxa (*C*. *peruviana*, *C*. *granulata*, *C*. *tosana*) were used for comparison. *Calypogeia arguta and C*. *sullivantii* were used as an outgroup. Bootstrap values above 50% are given above the branches.
(PDF)

S2 Fig Neighbor-joining trees of the *Calypogeia azurea* complex. The results of the ABGD analysis of individual loci are represented by colored stripes on the right side of the diagram. The first bar of the diagram represents the initial partition, and the second bar represents the recursive partition. The related taxa (*C*. *peruviana*, *C*. *granulata*, *C*. *aeruginosa*, *C*. *lunata* and *C*. *tosana*) were used for comparison. *Calypogeia arguta* and *C*. *sullivantii* were used as an outgroup. Bootstrap values above 50% are given above the branches.
(PDF)

S1 Table Collection details and GenBank accession numbers of the studied *Calypogeia* samples. *Samples from the herbarium collection, ^a-c^ references to GenBank sequences. Oil bodies ^1^ –based on the authors’ observations, ^2^ –based on herbarium data. Shaded lines indicate GenBank samples, and shaded cells indicate sequences from the authors’ previous work.
(DOCX)

S2 Table Primer sequences for amplification of *Calypogeia* specimens in the present study. (DOCX)

S3 Table Species specific sites for *C*. *azurea* s. str., *C*. *azurea*–NA, *C*. *sinensis* and *C*. *orientalis*. The related taxa (*C*. *peruviana*, *C*. *granulata*, *C*. *tosana*) were used for comparison.
(XLSX)

S4 Table Descriptive statistics for *C*. *azurea* s. str., *C*. *azurea*–NA, *C*. *sinensis* and *C*. *orientalis*. The means, minimum and maximum values of 47 quantitative traits were calculated according to Buczkowska (2004), N—number of measurements.
(PDF)

S1 Appendix The key to *Calypogeia* with blue oil bodies. (PDF)
