# Supplementary material for: Does Calypogeia azurea (Calypogeiaceae, Marchantiophyta) occur outside Europe? Molecular and morphological evidence
Source: PLoS One. 2018 Oct 10;13(10):e0204561. doi: 10.1371/journal.pone.0204561 (PMC6179228; doi:10.1371/journal.pone.0204561)
Supplement: S1 Fig — Neighbor joining (A) and maximum parsimony (B) consensus trees of the Calypogeia azurea complex. The analyses were performed separately for a combined plastid loci and nuclear ITS2. Only the accessions with complete sequences of all loci were included in the analysis. The related taxa (C. peruviana, C. granulata, C. tosana) were used for comparison. Calypogeia arguta and C. sullivantii were used as an outgroup. Bootstrap values above 50% are given above the branches. (PDF) [file pone.0204561.s001.pdf]

S1 Fig. Neighbor joining (A) and maximum parsimony (B) consensus trees of the *Calypogeia azurea* complex. The analyses were performed separately for a combined plastid loci and nuclear ITS2. Only the accessions with complete sequences of all loci were included in the analysis. The related taxa (*C. peruviana*, *C. granulata*, *C. tosana*) were used for comparison. *Calypogeia arguta and C. sullivantii* were used as an outgroup. Bootstrap values above 50% are given above the branches.

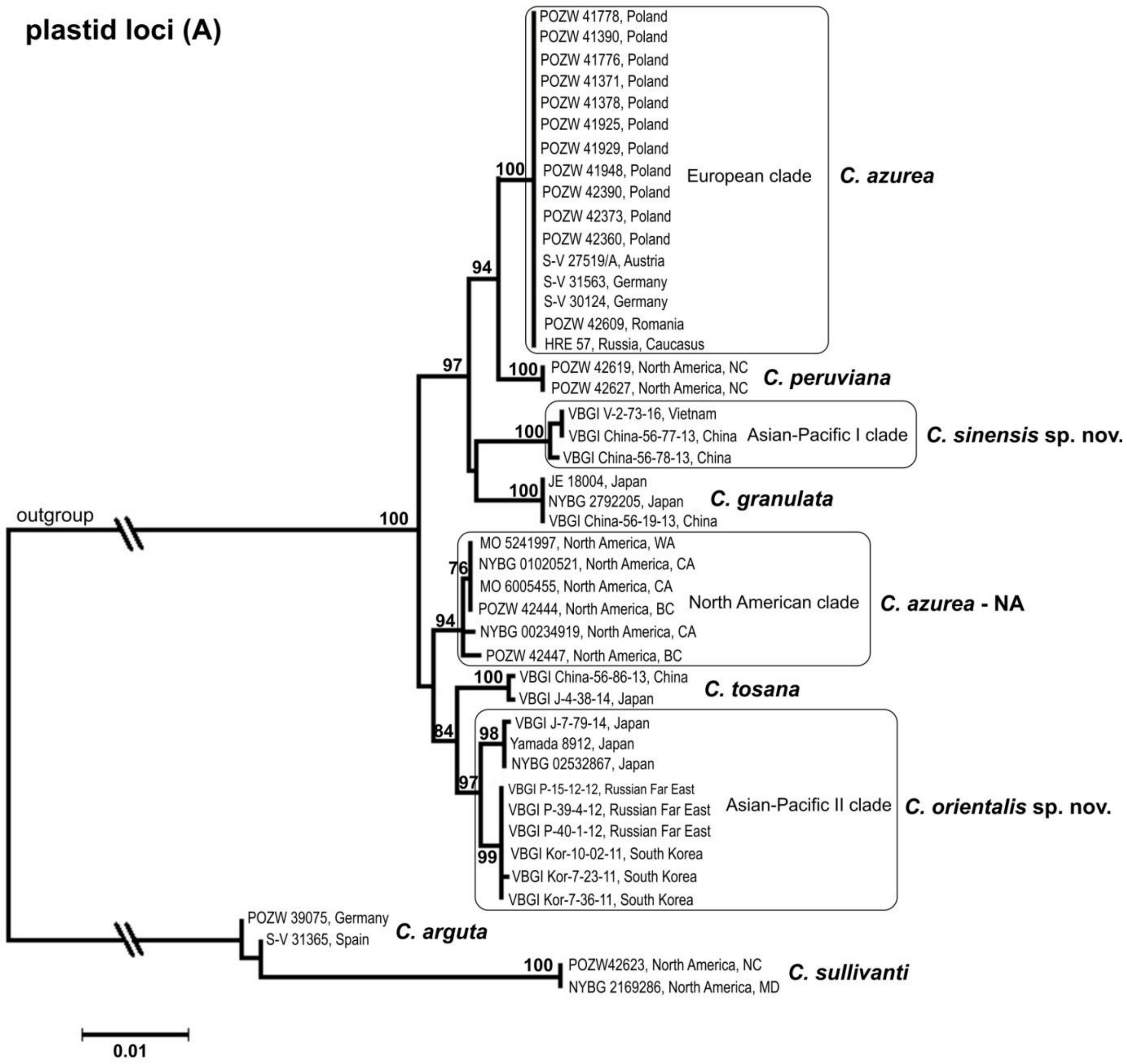

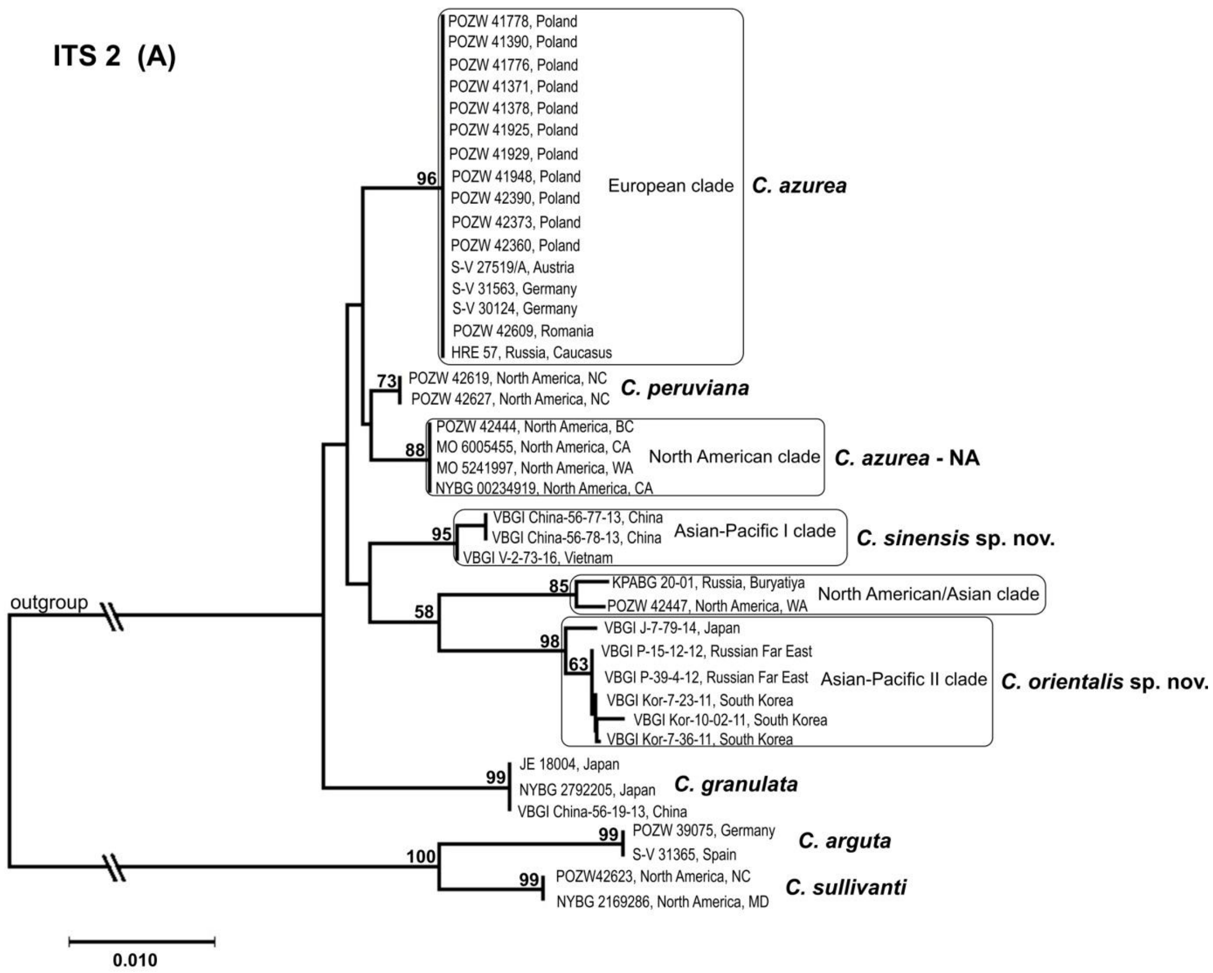
ITS 2 (A)
POZW 41778, Poland
POZW 41390, Poland
POZW 41776, Poland
POZW 41371, Poland
POZW 41378, Poland
POZW 41925, Poland
POZW 41929, Poland
POZW 41948, Poland
POZW 42390, Poland
POZW 42373, Poland
POZW 42360, Poland
S-V 27519/A, Austria
S-V 31563, Germany
S-V 30124, Germany
POZW 42609, Romania
HRE 57, Russia, Caucasus
96
European clade
C. azurea
73
POZW 42619, North America, NC
POZW 42627, North America, NC
C. peruviana
POZW 42444, North America, BC
MO 6005455, North America, CA
MO 5241997, North America, WA
NYBG 00234919, North America, CA
88
North American clade
C. azurea - NA
VBGI China-56-77-13, China
VBGI China-56-78-13, China
VBGI V-2-73-16, Vietnam
95
Asian-Pacific I clade
C. sinensis sp. nov.
85
KPABG 20-01, Russia, Buryatiya
POZW 42447, North America, WA
North American/Asian clade
outgroup
58
VBGI J-7-79-14, Japan
98
VBGI P-15-12-12, Russian Far East
63
VBGI P-39-4-12, Russian Far East
VBGI Kor-7-23-11, South Korea
VBGI Kor-10-02-11, South Korea
VBGI Kor-7-36-11, South Korea
Asian-Pacific II clade
C. orientalis sp. nov.
JE 18004, Japan
99
NYBG 2792205, Japan
VBGI China-56-19-13, China
C. granulata
99
POZW 39075, Germany
S-V 31365, Spain
C. arguta
100
99
POZW42623, North America, NC
NYBG 2169286, North America, MD
C. sullivanti
0.010

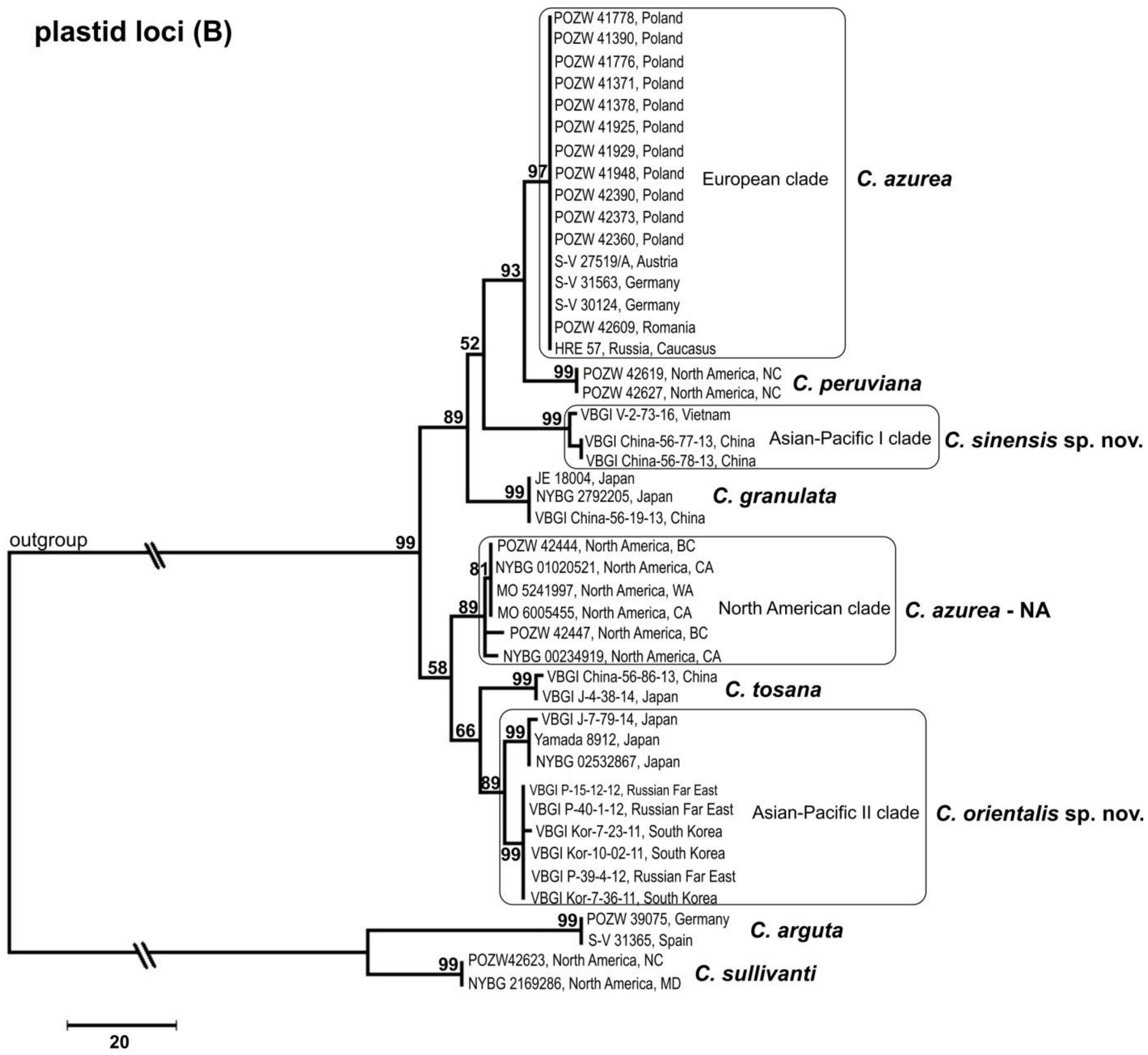
plastid loci (B)
POZW 41778, Poland
POZW 41390, Poland
POZW 41776, Poland
POZW 41371, Poland
POZW 41378, Poland
POZW 41925, Poland
POZW 41929, Poland
POZW 41948, Poland
POZW 42390, Poland
POZW 42373, Poland
POZW 42360, Poland
S-V 27519/A, Austria
S-V 31563, Germany
S-V 30124, Germany
POZW 42609, Romania
HRE 57, Russia, Caucasus
European clade
C. azurea
POZW 42619, North America, NC
POZW 42627, North America, NC
C. peruviana
VBGI V-2-73-16, Vietnam
VBGI China-56-77-13, China
VBGI China-56-78-13, China
Asian-Pacific I clade
C. sinensis sp. nov.
JE 18004, Japan
NYBG 2792205, Japan
VBGI China-56-19-13, China
C. granulata
outgroup
POZW 42444, North America, BC
NYBG 01020521, North America, CA
MO 5241997, North America, WA
MO 6005455, North America, CA
POZW 42447, North America, BC
NYBG 00234919, North America, CA
North American clade
C. azurea - NA
VBGI China-56-86-13, China
VBGI J-4-38-14, Japan
C. tosana
VBGI J-7-79-14, Japan
Yamada 8912, Japan
NYBG 02532867, Japan
VBGI P-15-12-12, Russian Far East
VBGI P-40-1-12, Russian Far East
VBGI Kor-7-23-11, South Korea
VBGI Kor-10-02-11, South Korea
VBGI P-39-4-12, Russian Far East
VBGI Kor-7-36-11, South Korea
Asian-Pacific II clade
C. orientalis sp. nov.
POZW 39075, Germany
S-V 31365, Spain
C. arguta
POZW42623, North America, NC
NYBG 2169286, North America, MD
C. sullivanti
97
93
52
89
99
81
89
58
66
89
20

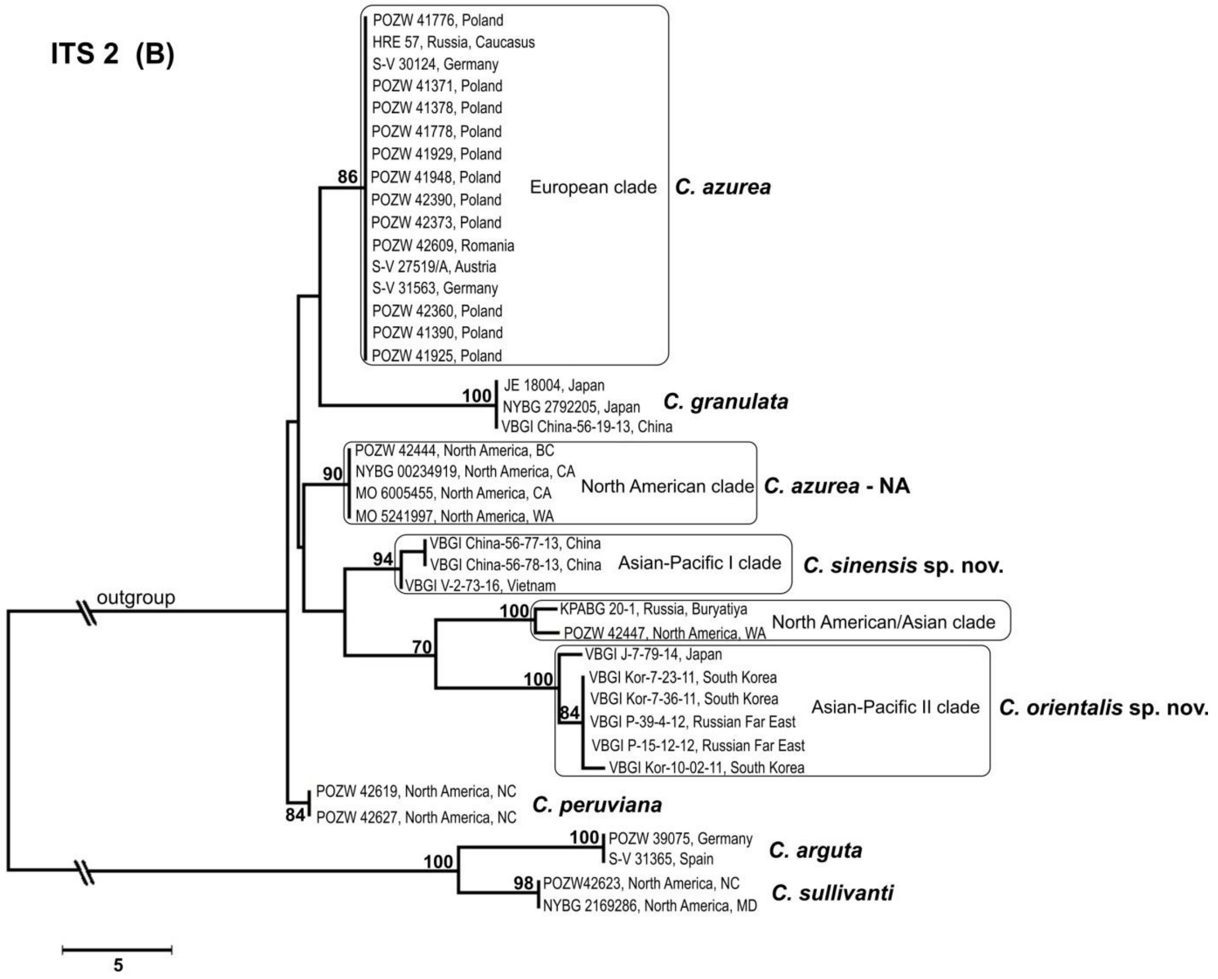
ITS 2 (B)
POZW 41776, Poland
HRE 57, Russia, Caucasus
S-V 30124, Germany
POZW 41371, Poland
POZW 41378, Poland
POZW 41778, Poland
POZW 41929, Poland
POZW 41948, Poland
POZW 42390, Poland
POZW 42373, Poland
POZW 42609, Romania
S-V 27519/A, Austria
S-V 31563, Germany
POZW 42360, Poland
POZW 41390, Poland
POZW 41925, Poland
86
European clade
C. azurea
JE 18004, Japan
NYBG 2792205, Japan
VBGI China-56-19-13, China
100
C. granulata
POZW 42444, North America, BC
NYBG 00234919, North America, CA
MO 6005455, North America, CA
MO 5241997, North America, WA
90
North American clade
C. azurea - NA
VBGI China-56-77-13, China
VBGI China-56-78-13, China
VBGI V-2-73-16, Vietnam
94
Asian-Pacific I clade
C. sinensis sp. nov.
outgroup
KPABG 20-1, Russia, Buryatiya
POZW 42447, North America, WA
100
North American/Asian clade
70
VBGI J-7-79-14, Japan
VBGI Kor-7-23-11, South Korea
VBGI Kor-7-36-11, South Korea
VBGI P-39-4-12, Russian Far East
VBGI P-15-12-12, Russian Far East
VBGI Kor-10-02-11, South Korea
100
84
Asian-Pacific II clade
C. orientalis sp. nov.
POZW 42619, North America, NC
POZW 42627, North America, NC
84
C. peruviana
POZW 39075, Germany
S-V 31365, Spain
100
100
C. arguta
POZW42623, North America, NC
NYBG 2169286, North America, MD
98
C. sullivanti
5
