# Supplementary material for: Does Calypogeia azurea (Calypogeiaceae, Marchantiophyta) occur outside Europe? Molecular and morphological evidence
Source: PLoS One. 2018 Oct 10;13(10):e0204561. doi: 10.1371/journal.pone.0204561 (PMC6179228; doi:10.1371/journal.pone.0204561)
Supplement: S2 Fig — The results of the ABGD analysis of individual loci are represented by colored stripes on the right side of the diagram. The first bar of the diagram represents the initial partition, and the second bar represents the recursive partition. The related taxa (C. peruviana, C. granulata, C. aeruginosa, C. lunata and C. tosana) were used for comparison. Calypogeia arguta and C. sullivantii were used as an outgroup. Bootstrap values above 50% are given above the branches. (PDF) [file pone.0204561.s002.pdf]

S2 Fig. Neighbor-joining trees of the *Calypogeia azurea* complex. The results of the ABGD analysis of individual loci are represented by colored stripes on the right side of the diagram. The first bar represents the initial partition, and the second bar represents the recursive partition. The related taxa (*C. peruviana*, *C. granulata*, *C. aeruginosa*, *C. lunata* and *C. tosana*) were used for comparison. *Calypogeia arguta and C. sullivantii* were used as an outgroup. Bootstrap values above 50% are given above the branches.

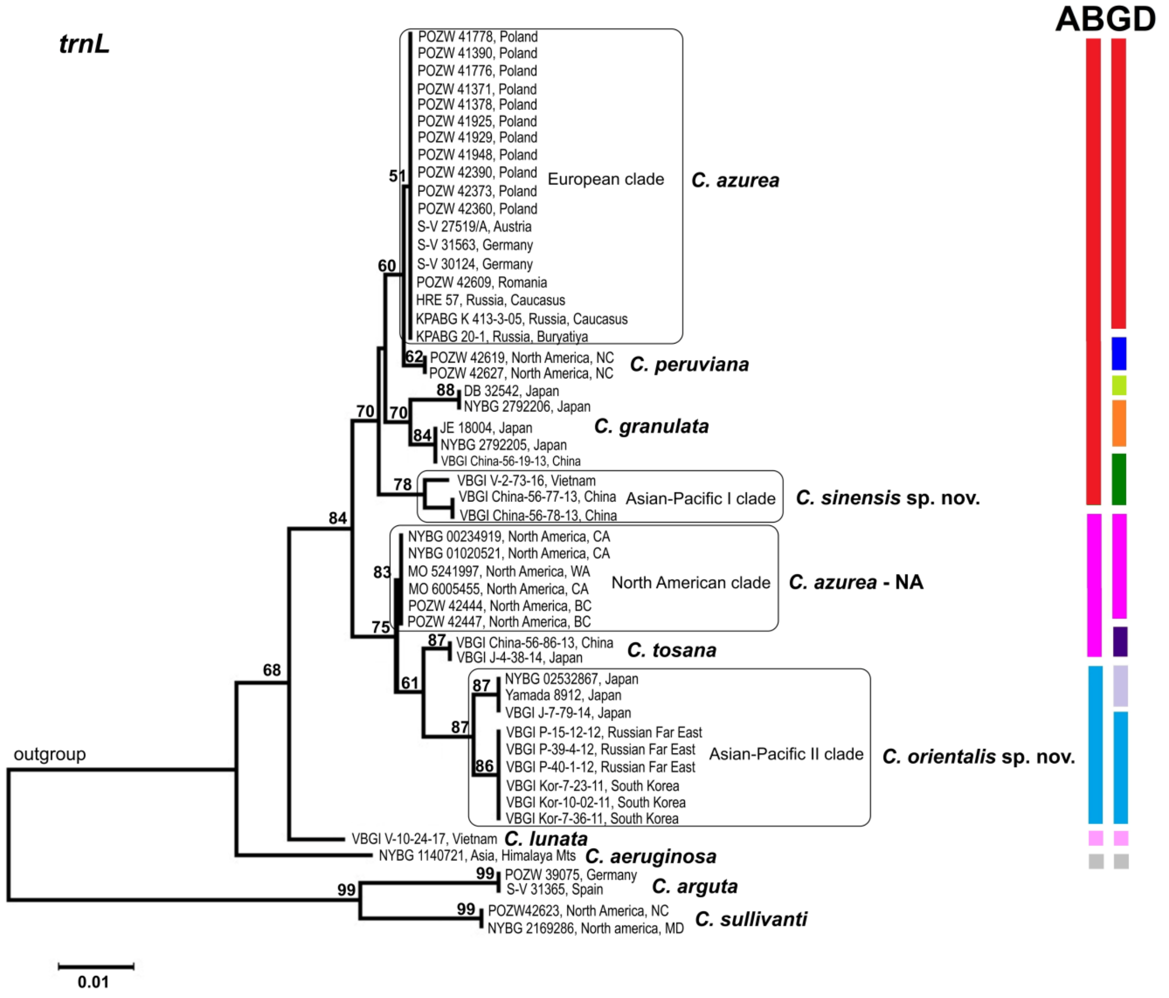

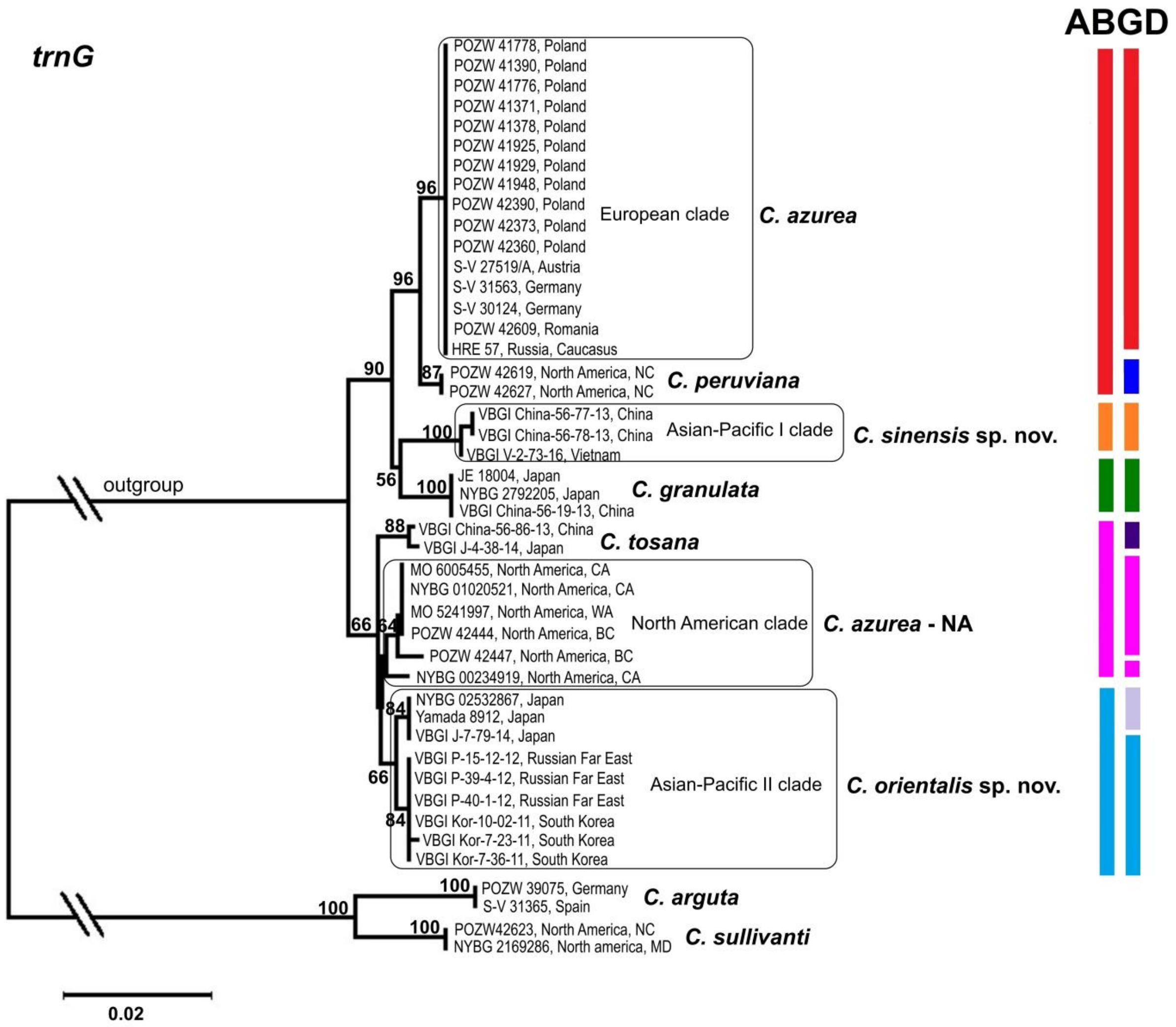

trnG
ABGD
POZW 41778, Poland
POZW 41390, Poland
POZW 41776, Poland
POZW 41371, Poland
POZW 41378, Poland
POZW 41925, Poland
POZW 41929, Poland
POZW 41948, Poland
POZW 42390, Poland
POZW 42373, Poland
POZW 42360, Poland
S-V 27519/A, Austria
S-V 31563, Germany
S-V 30124, Germany
POZW 42609, Romania
HRE 57, Russia, Caucasus
European clade
C. azurea
POZW 42619, North America, NC
POZW 42627, North America, NC
C. peruviana
VBGI China-56-77-13, China
VBGI China-56-78-13, China
VBGI V-2-73-16, Vietnam
Asian-Pacific I clade
C. sinensis sp. nov.
outgroup
JE 18004, Japan
NYBG 2792205, Japan
VBGI China-56-19-13, China
C. granulata
VBGI China-56-86-13, China
VBGI J-4-38-14, Japan
C. tosana
MO 6005455, North America, CA
NYBG 01020521, North America, CA
MO 5241997, North America, WA
POZW 42444, North America, BC
POZW 42447, North America, BC
NYBG 00234919, North America, CA
North American clade
C. azurea - NA
NYBG 02532867, Japan
Yamada 8912, Japan
VBGI J-7-79-14, Japan
VBGI P-15-12-12, Russian Far East
VBGI P-39-4-12, Russian Far East
VBGI P-40-1-12, Russian Far East
VBGI Kor-10-02-11, South Korea
VBGI Kor-7-23-11, South Korea
VBGI Kor-7-36-11, South Korea
Asian-Pacific II clade
C. orientalis sp. nov.
POZW 39075, Germany
S-V 31365, Spain
C. arguta
POZW42623, North America, NC
NYBG 2169286, North america, MD
C. sullivanti
96
96
90
87
100
56
100
88
66
64
84
66
84
100
100
100
0.02

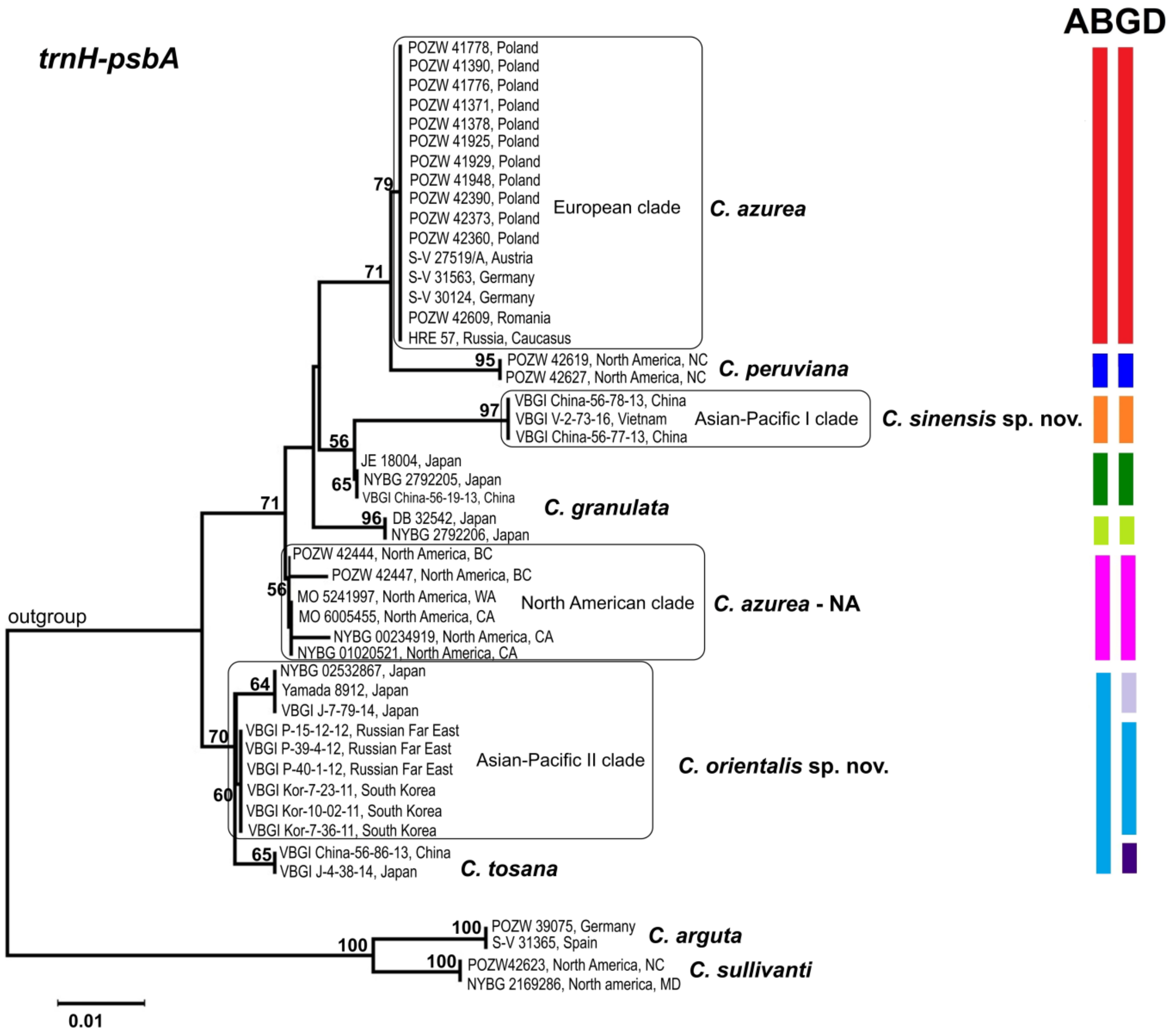
trnH-psbA
ABGD
POZW 41778, Poland
POZW 41390, Poland
POZW 41776, Poland
POZW 41371, Poland
POZW 41378, Poland
POZW 41925, Poland
POZW 41929, Poland
POZW 41948, Poland
POZW 42390, Poland
POZW 42373, Poland
POZW 42360, Poland
S-V 27519/A, Austria
S-V 31563, Germany
S-V 30124, Germany
POZW 42609, Romania
HRE 57, Russia, Caucasus
European clade
C. azurea
POZW 42619, North America, NC
POZW 42627, North America, NC
C. peruviana
VBGI China-56-78-13, China
VBGI V-2-73-16, Vietnam
VBGI China-56-77-13, China
Asian-Pacific I clade
C. sinensis sp. nov.
JE 18004, Japan
NYBG 2792205, Japan
VBGI China-56-19-13, China
DB 32542, Japan
NYBG 2792206, Japan
C. granulata
POZW 42444, North America, BC
POZW 42447, North America, BC
MO 5241997, North America, WA
MO 6005455, North America, CA
NYBG 00234919, North America, CA
NYBG 01020521, North America, CA
North American clade
C. azurea - NA
outgroup
NYBG 02532867, Japan
Yamada 8912, Japan
VBGI J-7-79-14, Japan
VBGI P-15-12-12, Russian Far East
VBGI P-39-4-12, Russian Far East
VBGI P-40-1-12, Russian Far East
VBGI Kor-7-23-11, South Korea
VBGI Kor-10-02-11, South Korea
VBGI Kor-7-36-11, South Korea
Asian-Pacific II clade
C. orientalis sp. nov.
VBGI China-56-86-13, China
VBGI J-4-38-14, Japan
C. tosana
POZW 39075, Germany
S-V 31365, Spain
C. arguta
POZW42623, North America, NC
NYBG 2169286, North america, MD
C. sullivanti
79
71
95
97
56
65
96
71
56
64
70
60
65
100
100
100
0.01

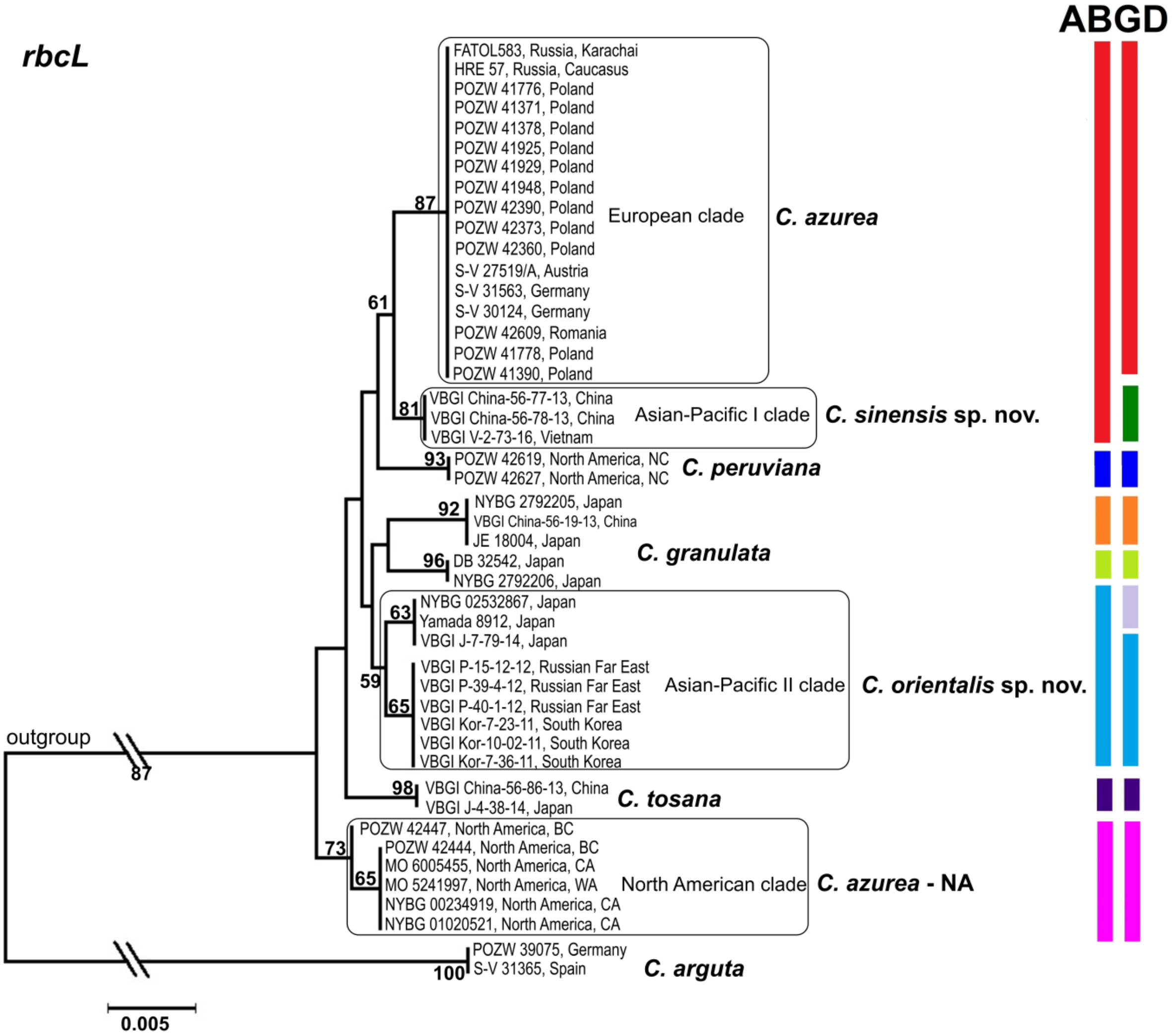
rbcL
ABGD
FATOL583, Russia, Karachai
HRE 57, Russia, Caucasus
POZW 41776, Poland
POZW 41371, Poland
POZW 41378, Poland
POZW 41925, Poland
POZW 41929, Poland
POZW 41948, Poland
POZW 42390, Poland
POZW 42373, Poland
POZW 42360, Poland
S-V 27519/A, Austria
S-V 31563, Germany
S-V 30124, Germany
POZW 42609, Romania
POZW 41778, Poland
POZW 41390, Poland
European clade
C. azurea
87
61
VBGI China-56-77-13, China
VBGI China-56-78-13, China
VBGI V-2-73-16, Vietnam
81
Asian-Pacific I clade
C. sinensis sp. nov.
93
POZW 42619, North America, NC
POZW 42627, North America, NC
C. peruviana
92
NYBG 2792205, Japan
VBGI China-56-19-13, China
JE 18004, Japan
96
DB 32542, Japan
NYBG 2792206, Japan
C. granulata
63
NYBG 02532867, Japan
Yamada 8912, Japan
VBGI J-7-79-14, Japan
59
65
VBGI P-15-12-12, Russian Far East
VBGI P-39-4-12, Russian Far East
VBGI P-40-1-12, Russian Far East
VBGI Kor-7-23-11, South Korea
VBGI Kor-10-02-11, South Korea
VBGI Kor-7-36-11, South Korea
Asian-Pacific II clade
C. orientalis sp. nov.
outgroup
87
98
VBGI China-56-86-13, China
VBGI J-4-38-14, Japan
C. tosana
73
65
POZW 42447, North America, BC
POZW 42444, North America, BC
MO 6005455, North America, CA
MO 5241997, North America, WA
NYBG 00234919, North America, CA
NYBG 01020521, North America, CA
North American clade
C. azurea - NA
POZW 39075, Germany
S-V 31365, Spain
100
C. arguta
0.005
