# Supplementary material for: Does Calypogeia azurea (Calypogeiaceae, Marchantiophyta) occur outside Europe? Molecular and morphological evidence
Source: PLoS One. 2018 Oct 10;13(10):e0204561. doi: 10.1371/journal.pone.0204561 (PMC6179228; doi:10.1371/journal.pone.0204561)
Supplement: S1 Table — *Samples from the herbarium collection, a-c references to GenBank sequences. Oil bodies 1 –based on the authors’ observations, 2 –based on herbarium data. Shaded lines indicate GenBank samples, and shaded cells indicate sequences from the authors’ previous work. (DOCX) [file pone.0204561.s003.docx]

**S1 Table.** **Collection details** **and GenBank accession numbers of the studied *Calypogeia* samples.** *Samples from the herbarium collection, ^a-c^ references to GenBank sequences. Oil bodies ^1^ – based on the authors’ observations, ^2^ – based on herbarium data. Shaded lines indicate GenBank samples, and shaded cells indicate sequences from the authors’ previous work.

| No. | Locality | Collector | Herbarium No. | Oil bodies | Accession number^1^ | | | | |
| --- | --- | --- | --- | --- | --- | --- | --- | --- | --- |
|  |  |  |  |  | *rbcL* | *trnL* | *trnG* | *trnH-psbA* | ITS2 |
|  | ***C. azurea*** |  |  |  |  |  |  |  |  |
| 1 | Europe, NE Poland, Warmińsko-Mazurskie Province, Lake Godle near Ełk, on wet humus soil | KB, AB | POZW 41778 | blue^1^ | MH367571 | MH367766 | MH367644 | MH367711 | MH367532 |
| 2 | Europe, S Poland, Tatra Mts, Rów Zakopiański at N base of Tatra Mts, Las Capowski forest, on soil, 971 m a.s.l. | AB, KB | POZW 41390 | blue^1^ | MH367572 | MH367767 | MH367645 | MH367712 | MH367533 |
| 3 | Europe, S Poland, Tatra Mts, Dolina Roztoki Valley, on soil, 1560 m a.s.l. | KB, AB | POZW 41776 | blue^1^ | MH367573 | MH367768 | MH367646 | MH367713 | MH367534 |
| 4 | Europe, S Poland, Tatra Mts, Sucha Woda Valley, Psia Trawka meadow, on soil, 1183 m a.s.l. | KB, AB | POZW 41371 | blue^1^ | MH367574 | MH367769 | MH367647 | MH367714 | MH367535 |
| 5 | Europe, S Poland, Tatra Mts, Dolina Pięciu Stawów Polskich Valley, N side of Czarny Staw, on soil, 1720 m a.s.l. | KB, AB | POZW 41378 | blue^1^ | MH367575 | MH367770 | MH367648 | MH367715 | MH367536 |
| 6 | Europe, SE Poland, Bieszczady Mts, W slope of Tarnica Mt., on humus soil, 1300 m a.s.l. | KB, BCh | POZW 41925 | blue^1^ | MH367576 | MH367771 | MH367649 | MH367716 | MH367537 |
| 7 | Europe, SE Poland, Bieszczady Mts, W slope of Rozsypaniec Wołosacki Mt., on humus soil, 1215 m a.s.l. | KB, BCh | POZW 41929 | blue^1^ | MH367577 | MH367772 | MH367650 | MH367717 | MH367538 |
| 8 | Europe, SE Poland, Bieszczady Mts, Wetlina, valley of Górna Solinka stream, on soil, 720 m a.s.l. | KB, BCh | POZW 41948 | blue^1^ | MH367578 | MH367773 | MH367651 | MH367718 | MH367539 |
| 9 | Europe, SE Poland, Gorce Mts, Ochotnica Dolna Kudowy, on soil, 655 m a.s.l. | KB, BCh | POZW 42390 | blue^1^ | MH367579 | MH367774 | MH367652 | MH367719 | MH367540 |
| 10 | Europe, SE Poland, Beskid Sądecki Mts, Potok Lipowiec stream, on soil, 520 m a.s.l. | KB, BCh | POZW 42373 | blue^1^ | MH367580 | MH367775 | MH367653 | MH367720 | MH367541 |
| 11 | Europe, SE Poland, Beskid Sądecki Mts, Potok Biały stream, on soil, 585 m a.s.l. | KB, BCh | POZW 42360 | blue^1^ | MH367581 | MH367776 | MH367654 | MH367721 | MH367542 |
| 12* | Europe, Austria, Kärnten, Karnische Alpen, Obers Gailtail, 1200 m a.s.l. | AS-V | S-V 27519/A | blue^1^ | MH367582 | MH367777 | MH367655 | MH367722 | MH367543 |
| 13* | Europe, Germany , Bayerische Alpen, Berchtesgadener Land, 785 m a.s.l. | AS-V | S-V 31563 | blue^1^ | MH367583 | MH367778 | MH367656 | MH367723 | MH367544 |
| 14* | Europe, Germany, Baden-Württemberg, Alpenvorland, Kreis Sigmaringen, 610 m a.s.l. | AS-V | S-V 30124 | blue^1^ | MH367584 | MH367779 | MH367657 | MH367724 | MH367545 |
| 15 | Europe, Romania , Arpaşu de Sus, Sibiu County | SS | POZW 42609 | blue^1^ | MH367585 | MH367780 | MH367658 | MH367725 | MH367546 |
| 16* | Europe, Russia, Northern Caucasus, Karachai - Cherkessk Republic, Teberda River, 2540 a.s.l. | NAK, ANS | HRE 57 | blue^1^ | MH367586 | MH367781 | MH367659 | MH367726 | MH367547 |
| 17^a^ | Europe, Russia, Caucasus | NAK | KPABG K413-3-05 | no data available | **⎯** | JF421610 | **⎯** | **⎯** | **⎯** |
| 18^b^ | Asia, Russia, Republic of Buryatiya (isolate c53) | NAK | KPABG 20-01 | no data available | **⎯** | JX630063 | **⎯** | **⎯** | JX629936 |
| 19^c^ | Asia, Russia, Northern Caucasus, Karachai - Cherkessk Republic (isolate FATOL583) | NAK | F | no data available | KC297120 | **⎯** | **⎯** | **⎯** | **⎯** |
| 20 | North America, Canada, British Columbia, Vancouver Island, San Juan Ridge - Jordan Ridge area, along FR JR300 E of Jordan River Main Rd., on wet shaded peat in depression in sloping fen. 910 m a.s.l. 1133B/7 | BA | POZW 42447 | blue^1^ | MH367587 | MH367782 | MH367660 | MH367727 | MH367548 |
| 21 | North America, Vancouver Island, Mt. Washington area at the end of Strathcona Pkwy, Paradise Meadows, 1139a on mineral soil on uprooted tree in conifer forest. 1090 m a.s.l, | BA | POZW 42444 | blue^1^ | MH367588 | MH367783 | MH367661 | MH367728 | MH367549 |
| 22* | North America, USA, California Modoc Plateau, North Warner Mountains, on organic debris and creek bank | WTD | MO 6005455 | blue^2^ | MH367589 | MH367784 | MH367662 | MH367729 | MH367550 |
| 23* | North America, USA, Washington, Austin Pass, Mt. Baker, Whatcom County, ca. 1800 m a.s.l. | WH | MO 5241997 | not determined | MH367590 | MH367785 | MH367663 | MH367730 | MH367551 |
| 24* | North America, USA, California, Humboldt Country, North Coast Ranges, on decorticated log | WTD | NYBG 00234919 | blue^2^ | MH367591 | MH367786 | MH367664 | MH367731 | MH367552 |
| 25* | North America, USA, California, Modoc Plateau, North Warner Mountains, on organic debris and creek bank | WTD | NYBG 01020521 | blue^2^ | MH367592 | MH367787 | MH367665 | MH367732 | ⎯ |
|  | ***C. orientalis sp.nov.*** |  |  |  |  |  |  |  |  |
| 26 | Pacific Asia, Russian Far East, Primorsy Territory, Shkotovsky District, Livadijsky Range, N slope of Litovka Mt., 550 m a.s.l. | VAB | VBGI P-15-12-12 | dark blue^1^ | MH367593 | MH367788 | MH367666 | MH367733 | MH367553 |
| 27 | Pacific Asia, Russian Far East, Primorsy Territory, Shkotovsky District, Livadijsky Range, N slope of Litovka Mt., 450 m a.s.l. | VAB | VBGI P-39-4-12 | dark blue^1^ | MH367594 | MH367789 | MH367667 | MH367734 | MH367554 |
| 28 | Pacific Asia, Russian Far East, Primorsy Territory, Shkotovsky District, Livadijsky Range, N slope of Litovka Mt., 400 m a.s.l. | VAB | VBGI P-40-1-12 | dark blue^1^ | MH367595 | MH367790 | MH367668 | MH367735 | **⎯** |
| 29 | Pacific Asia, South Korea, Seorak Mt. National Park in Kongwon Prov. Bonjeong Temple area, 1250 m a.s.l. | VAB | VBGI Kor-7-23-11 | dark blue^1^ | MH367596 | MH367791 | MH367669 | MH367736 | MH367555 |
| 30 | Pacific Asia, South Korea, Seorak Mt. National Park in Kongwon Prov. Jungcheong Peak, 1550 m a.s.l. | VAB | VBGI Kor-10-02-11 | dark blue^1^ | MH367597 | MH367792 | MH367670 | MH367737 | MH367556 |
| 31 | Pacific Asia, South Korea, Seorak Mt. National Park in Kongwon Prov. Bonjeong Temple area, 1250 m a.s.l. | VAB | VBGI Kor-7-36-11 | dark blue^1^ | MH367598 | MH367793 | MH367671 | MH367738 | MH367557 |
| 32 | Pacific Asia, Japan, Kyushu Isl. Fukuoka Pref. Tagama-gun, Soedamachi, Hiko-san Mt. 770 m a.s.l. | VAB | VBGI J-7-79-14 | deep blue^1^ | MH367599 | MH367794 | MH367672 | MH367739 | MH367558 |
| 33* | Pacific Asia, Japan, Honshu, Ibaraki Pref., Kuji-gun, Mt. Yamizo, on decaying log AT stream, on decaying log at stream, 720m a.s.l. | MH | NYBG 02532867 | not determined | MH367600 | MH367795 | MH367673 | MH367740 | **⎯** |
| 34* | Pacific Asia, Japan, Mie Pref.: Aota, Iitaka-cho, on decayed woods under forest, ca. 500 m a.s.l. | KY | Yamada 8912 | not determined | MH367601 | MH367796 | MH367674 | MH367741 | **⎯** |
|  | ***C. sinensis sp.nov.*** |  |  |  |  |  |  |  |  |
| 35 | Pacific Asia, China, Guizhou Proivince, Duyun Municipality, Doupeng Mountains, Xiniu Waterfall area, 1300 m a.s.l. | VAB | VBGI China-56-77-13 | deep blue^1^ | MH367602 | MH367797 | MH367675 | MH367742 | MH367559 |
| 36 | Pacific Asia, China, Guizhou Proivince, Duyun Municipality, Doupeng Mountains, Xiniu Waterfall area, 1300 m a.s.l. | VAB | VBGI China-56-78-13 | dark blue^1^ | MH367603 | MH367798 | MH367676 | MH367743 | MH367560 |
| 37 | Pacific Asia, Vietnam, Lao Cai Province, SaPa, Phan Xi Pan National Park, mesic decaying wood, in part shade, 1900-2100 m a.s.l. | VAB | VBGI V-2-73-16 | dark blue^1^ | MH367604 | MH367799 | MH367677 | MH367744 | MH367561 |
|  | ***C. peruviana*** |  |  |  |  |  |  |  |  |
| 38 | North America, USA, North Carolina, Macon Co., Chattooga River Gorge trail from government bridge, sandy trail bank through Rhododendron thicket in cove forest, 740 m a.s.l. 1132c/4 | BA | POZW 42619 | blue^1^ | MH367605 | MH367800 | MH367678 | MH367745 | MH367562 |
| 39 | North America, USA, North Carolina, Macon Co., Chattooga River Gorge trail from government bridge, Wet stream bank in Rhododendron maximum & Leucothoe thicket, 740 m a.s.l. | BA | POZW 42627 | blue^1^ | MH367606 | MH367801 | MH367679 | MH367746 | MH367563 |
|  | ***C. granulata*** |  |  |  |  |  |  |  |  |
| 40* | Pacific Asia, Japan, Kuroyama, Saitama Pref., on moist rocks near watter fall, ca. 500 m a.s.l. | HI | JE 18004  type | blue or grayish blue | MH367607 | MH367802 | MH367680 | MH367747 | MH367564 |
| 41* | Pacific Asia, Japan, Kuroyama, Iruma-gun, Saitama Pref. (Type locality), on very moist rocks near stream, ca 500 m a.s.l. (Type locality) | HI | NYGB 2792205 | not determined | MH367608 | MH367803 | MH367681 | MH367748 | MH367565 |
| 42 | Pacific Asia, China, Guizhou Proivince, Duyun Municipality, Doupeng Mountains, Xiniu Waterfall area, 1300 m a.s.l. | VAB | VBGI China-56-19-13 | gray | MH367609 | MH367804 | MH367682 | MH367749 | MH367566 |
| 43* | Pacific Asia, Japan, Kyushu, (Yakushima Isl.) Kogoshima-ken, on moist rock face at open site, 1380 m a.s.l. | TY | DB 32542 | not determined | MH367610 | MH367805 | **⎯** | MH367750 | **⎯** |
| 44* | Pacific Asia, Japan, Kyushu, (Yakushima Isl.) Kogoshima-ken, on moist rock face at open site, 1380 m a.s.l. | TY | NYGB 2792206 | not determined | MH367611 | MH367806 | **⎯** | MH367751 | **⎯** |
|  | ***C. tosana*** |  |  |  |  |  |  |  |  |
| 45 | Pacific Asia, China, China, Guizhou Proivince, Duyun Municipality, Doupeng Mountains, Xiniu Waterfall area, wet clif in open placein spray zone of waterfall, 1300 m a.s.l. | VAB | VBGI China-56-86-13 | not determined | MH367612 | MH367807 | MH367683 | MH367752 | **⎯** |
| 46 | Pacific Asia, Japan, Kyushu Isl., Fukuoka Pref., Tagama-gun, Soedamach, Hiko-san Mt, moist humus in partial shade, on slope, 570 m a.s.l. | VAB | VBGI J-4-38-14 | not determined | MH367613 | MH367808 | MH367684 | MH367753 | **⎯** |
|  | ***C. lunata*** |  |  |  |  |  |  |  |  |
| 47 | Pacific Asia, Vietnam | VAB | VBGI V-10-24-17 | not determined | **⎯** | MH367809 | **⎯** | **⎯** | **⎯** |
|  | ***C. aeruginosa*** |  |  |  |  |  |  |  |  |
| 48* | Asia, India, Himalaya Mts, Lachen in North Sikkim.** | JDH | NYGB 1140721  Isotype? | not determined | **⎯** | MH367810 | **⎯** | **⎯** | **⎯** |
|  | ***C. sphagnicola* f. *paludosa*** |  |  |  |  |  |  |  |  |
| 49 | Europe, S Poland, Tatra Mts, E slope of Mt. Żółta Turnia, sphagnum-polytrichum hummocks, 1687 m a.s.l. | KB, AB | POZW 41174 | colorless | MH367616 | JQ658795 | JQ658778 | JF776829 | **⎯** |
| 50 | Europe, S Poland, Tatra Mts, peat bog Toporowy Staw Wyżni, 1110 m a.s.l. | KB, AB | POZW 41148 | colorless | MH367617 | JQ658796 | JQ658779 | JF776831 | **⎯** |
| 51 | Europe, S Poland, Tatra Mts, Pańszczyca Valley, peat bog Wielka Pańszczycka Młaka, 1274 m a.s.l. | KB, AB | POZW 42277 | colorless | MH367618 | JQ658797 | JQ658780 | JF776830 | **⎯** |
| 52 | Europe, S Poland, Tatra Mts, N slope of Mt. Ornak, sphagnum-polytrichum hummocks, 1700 m a.s.l. | KB, AB | POZW 41722 | colorless | MH367619 | JQ658798 | JQ658781 | JF776832 | **⎯** |
|  | ***C. sphagnicola* f. *sphagnicola*** |  |  |  |  |  |  |  |  |
| 53 | Europe, NW Poland, Pomorskie Province, peat mat in the littoral zone of Lake Wałachy near Wdzydze | KB, AB | POZW 42284 | colorless | MH367620 | JQ658790 | JQ658773 | JF776837 | **⎯** |
| 54 | Europe, NW Poland, Pomorskie Province, peat mat in the littoral zone of Lake Małe Katarzynki near Borowy Młyn | KB, AB | POZW 42245 | colorless | MH367621 | JQ658791 | JQ658774 | JF776836 | **⎯** |
| 55 | Europe, NW Poland, Pomorskie Province, Lake Czyste peat bog near Płocice | KB, AB | POZW 42266 | colorless | MH367622 | JQ658793 | JQ658776 | JF776838 | **⎯** |
| 56 | Europe, NE Poland, Warmińsko-Mazurskie Province, peat mat in the littoral zone of Godle lake near Ełk | KB, AB | POZW 41711 | colorless | MH367623 | JQ658794 | JQ658777 | JF776839 | **⎯** |
|  | ***C. suecica*** |  |  |  |  |  |  |  |  |
| 57 | Europe, S Poland, Beskid Żywiecki Mts, Mt. Babia Góra, 1190 m a.s.l. | JS, KB | POZW 39500 | colorless | MH367624 | MH367815 | MH367689 | JF776835 | **⎯** |
| 58 | Europe, S Poland, Tatra Mts, stream near Lake Toporowy Staw Wyżni, 1110 m a.s.l. | KB, AB | POZW 41727 | colorless | MH367625 | MH367816 | MH367690 | JF776834 | **⎯** |
| 59 | Europe, SE Poland, Bieszczady Mts, Górna Solinka Valley, 772 m a.s.l. | KB, BCh | POZW 41936 | colorless | MH367626 | MH367817 | MH367691 | JF776833 | **⎯** |
| 60 | Europe, S Poland, Tatra Mts, Dolona Kościeliska Valley, Pisaniarski Żleb, 1200 m a.s.l. | KB, AB | POZW 41344 | colorless | MH367627 | MH367818 | MH367692 | MH367758 | **⎯** |
|  | ***C. neesiana*** |  |  |  |  |  |  |  |  |
| 61 | Europe, S Poland, Tatra Mts, N slope of Mt. Ornak, sphagnum-polytrichum hummocks, 1680 m a.s.l. | KB, AB | POZW 41731 | colorless | MH367628 | MH367819 | MH367693 | JF776844 | **⎯** |
| 62 | Europe, NE Poland, Warmińsko-Mazurskie Province, Mechacz Wielki peat bog | KB, AB | POZW 41735 | colorless | MH367629 | MH367820 | MH367694 | JF776845 | **⎯** |
| 63 | Europe, SE Poland, Bieszczady Mts, W slope of Mt. Rozsypaniec Wołosacki, 1204 m a.s.l. | KB, BCh | POZW 41927 | colorless | MH367630 | MH367821 | MH367695 | JF776846 | **⎯** |
| 64 | Europe, S Poland, Tatra Mts, E slope of Mt. Żółta Turnia, 1670 m a.s.l. | KB, AB | POZW 41358 | colorless | MH367631 | MH367822 | MH367696 | MH367759 | **⎯** |
|  | ***C. integristipula*** |  |  |  |  |  |  |  |  |
| 65 | Europe, NE Poland, Warmińsko-Mazurskie Province, Mechacz Wielki peat bog | KB, AB | POZW 41730 | colorless | MH367632 | MH367823 | MH367697 | JF776848 | **⎯** |
| 66 | Europe, SE Poland, Bieszczady Mts, W slope of Mt. Rozsypaniec Wołosacki, 1214 m a.s.l. | KB, BCh | POZW 41928 | colorless | MH367633 | MH367824 | MH367698 | JF776849 | **⎯** |
| 67 | Europe, NE Poland, Warmińsko-Mazurskie Province, Lake Godle near Ełk | KB, AB | POZW 41785 | colorless | MH367634 | MH367825 | MH367699 | MH367760 | **⎯** |
| 68 | Europe, NW Poland, Pomorskie Province, Staniszewskie Błota near Kartuzy | KB, AB | POZW 41188 | colorless | MH367635 | MH367826 | MH367700 | MH367761 | **⎯** |
|  | ***C. fissa*** |  |  |  |  |  |  |  |  |
| 69 | Europe, Germany, Bonn – Bad Godesberg, Annaberger Bachtal, pine forest | AS, DQ | POZW 39074 | colorless | MH367636 | MH367827 | MH367701 | MH367762 | **⎯** |
| 70 | Europe, W Poland, Lubuskie Province, Nabłotno forestry, humus in *Carici elongate-Alnetum* | SR | POZW 42437 | colorless | MH367637 | MH367828 | MH367702 | MH367763 | **⎯** |
| 71 | Europe, Germany, Baden-Württemberg, western Bodenseegebiet, Schienerberg-Nordabhang above Bankholzen, Öde Halde north from Langenmoos, 620 m a.s.l. | AS-V | S-V 25448 | colorless | MH367638 | MH367829 | MH367703 | MH367764 | **⎯** |
| 72 | Europe, Georgia, Ajaria, Batumi, in Horto Botanico, pars Australiae. | AA | OP 110668 | colorless | MH367639 | MH367830 | MH367704 | MH367765 | **⎯** |
|  | ***C. muelleriana*** |  |  |  |  |  |  |  |  |
| 73 | Europe, NE Poland, Warmińsko-Mazurskie Province, Lake Godle near Ełk | KB, AB | POZW 41708 | colorless | MH367640 | KF371552 | KF371604 | KF371578 | **⎯** |
| 74 | Europe, W Poland, Lubuskie Province, Starosiedle foresty | SR, KB | POZW 42323 | colorless | MH367641 | KF371556 | KF371608 | KF371582 | **⎯** |
| 75 | Europe, Germany, Baden-Württemberg, western Bodenseegebiet, Kreis Konstanz, Schiener-Berg-Nordabhang above Bankholzen, 630 m a.s.l. | AS-V | S-V 28577 | colorless | MH367642 | KF371560 | KF371612 | KF371586 | **⎯** |
| 76 | Europe, United Kingom, South Lancashire, in damp sandstone crevice of disused quarry wall | DAC | DC 1421 | colorless | MH367643 | KF371565 | KF371617 | KF371591 | **⎯** |
|  | ***outgroup*** |  |  |  |  |  |  |  |  |
|  | ***C. arguta*** |  |  |  |  |  |  |  |  |
| 77 | Europe, Germany, Bonn – Bad Godesberg, Annaberger Bachtal, pine forest | AS, DQ | POZW 39075 | colorless | MH367614 | MH367811 | MH367685 | MH367754 | MH367567 |
| 78* | Europe, Spain, Galicia, Province Ourense, Serra Do Xurés National Park | AS-V | S-V 31365 | colorless | MH367615 | MH367812 | MH367686 | MH367755 | MH367568 |
|  | ***C. sullivanti*** |  |  |  |  |  |  |  |  |
| 79 | North America, USA, North Carolina, Dry Falls | BA | POZW 42623 | slightly grayish | **⎯** | MH367813 | MH367687 | MH367756 | MH367569 |
| 80* | North America, USA, Maryland, Dorchester County, Rte 313, Sharptown | LTB | NYGB 2169286 | not determined | **⎯** | MH367814 | MH367688 | MH367757 | MH367570 |
|  | **Misidentified in herbarium as *C. azurea*, excluded from the further analyses** |  |  |  |  |  |  |  |  |
| 81* | ***C. fissa*** - North America, Canada, Nova Scotia, Yarmouth | JM | POZW 3337 | not determined | **⎯** | MH367831 | MH367705 | **⎯** | **⎯** |
| 82* | ***C. fissa*** - Europe, Ireland, Ballymount Bog, Calverstown | JST | POZW 3344 | not determined | **⎯** | MH367832 | MH367706 | **⎯** | **⎯** |
| 83* | ***C. fissa*** - Europe, Spain, Algeciras (Cádiz) Las Chorreras | CC | POZW 3345 | not determined | **⎯** | MH367833 | MH367707 | **⎯** | **⎯** |
| 84* | ***C. fissa*** - Europe, Hungary, Alsószölnök | LV | POZW 3347 | not determined | **⎯** | MH367834 | MH367708 | **⎯** | **⎯** |
| 85* | ***C. sphagnicola*** - Pacific Asia, Japan, Asakusa Mt., Tadami-cho, Fukushima Pref. on wet soil, 1400 m a.s.l. | HI | KRAM 50656 | not determined | **⎯** | **⎯** | MH367709 | **⎯** | **⎯** |
| 86* | ***C. muelleriana*** - North America, Canada, Cape Fife Trail, Naikoon Prov. Pk. Graham I. QCI, ca. 20-50 m a.s.l. | WH | MO 5241995 | not determined | **⎯** | MH367835 | MH367710 | **⎯** | **⎯** |

Collectors: AA – A. Abramovi; AB – Alina Bączkiewicz; ANS - A.N. Savchenko; AS – A. Sloga; AS-V – Alfons Schäfer-Verwimp; BA – Blanka Aguero; BCh – Błażej Chmielewska; CC – C. Casas; DK – D. Killmann; DAC – D. A. Callaghan; DQ – D. Quandt,; EF – E. Fischer; HI – H. Inoue; JDH - J.D. Hooker; JM – John Macoun; JST – J.S. Thomson; KB – Katarzyna Buczkowska; KY – Kohsaku Yamada; LTB – Lance T. Biechele; LV – L. Vajda; NAK – N.A. Konstantinova; SS – S. Ştefănut, TY – T. Yamaguchi; VAB - Vadim A. Bakalin, WTD - W. T. Doyle, WH - W. Hong, MH – M. Higuchi

Herbaria: S-V – Herb. Schäfer-Verwimp, POZW – Herbarium of Adam Mickiewicz University, HRE - Hepaticae Rossicae Exsiccate, FEB RAS (VBGI) - Bryophyte Herbarium of the Botanical Garden-Institute, KRAM - , MO - Missouri Botanical Garden Herbarium, NYBG – New York Botanical Garden Herbarium, F – Field Museum of Natural History, JE - Jena, OP – Musei Silesiensis Opava

**References:**

^a^Vilnet AA, Konstantinova NA, Troitsky AV. Taxonomical rearrangements of Solenostomataceae (Marchantiophyta) with description of a new family Endogemmataceae based on *trn*L-F cpDNA analysis. Folia Cryptog. Estonica, Fasc. 2011; 48, 125-133.

^b^Vilnet AA, Konstantinova NA, Troitsky AV. Molecular phylogeny and systematics of the suborder Cephaloziineae with special attention to the family Cephaloziaceae s.l. (Jungermanniales, Marchantiophyta). Arctoa. 2012; 21, 113-132.

^c^Bainard JD, Forrest LL, Goffinet B, Newmaster SG. Nuclear DNA content variation and evolution in liverworts. Mol Phylogenet Evol. 2013; 68, 619-627.

**Mitten W. Hepaticae Indiae Orientalis, An enumeration of the Hepaticae of East Indies. Journal of the Proceedings of the Linnean Society (Bot.). 1861;5, 89-128.
