# Supplementary material for: Does Calypogeia azurea (Calypogeiaceae, Marchantiophyta) occur outside Europe? Molecular and morphological evidence
Source: PLoS One. 2018 Oct 10;13(10):e0204561. doi: 10.1371/journal.pone.0204561 (PMC6179228; doi:10.1371/journal.pone.0204561)
Supplement: S2 Table — (DOCX) [file pone.0204561.s004.docx]

**S2 Table. Primer sequences of the amplification of *Calypogeia* specimens in the present study.**

| DNA Region | Primer name | Direction | Primer sequence (5’- 3’) | T°C | References |
| --- | --- | --- | --- | --- | --- |
| *rbcL-a* | a_f | F | ATGTCACCACAAACAGAGACTAAAGC | 60 | ^a^ Kress & Erickson (2007) |
|  | a_r | R | CTTCTGCTACAAATAAGAATCGATCTC |  |  |
| *trnL* | A | F | GGGGGTATGGCGAAATTGG | 62 | ^b^ Pacak & Szweykowska-Kulińska (2003) |
|  | B | R | TGG GGG TAG AGG GAC TTG |  |  |
| *trnG* | A | F | CGG GTA CGG GAA TCG AAC | 60 | ^b^ Pacak & Szweykowska-Kulińska (2003) |
|  | C | R | GCG GGT ATA GTT TAG TGG |  |  |
| *psbA- trnH* | psbA F | F | GTTATGCATGAACGTAATGCTC | 50 | ^c^ Sang et al. 1997 |
|  | trnH R | R | CGCGCATGGTGGATTCACAAATC |  |  |
| ITS2 | F | F | CGGATATCTTGGCTCTTG | 60 | ^d^ Sawicki et al. 2010 |
|  | R | R | CCGCTTAGTGATATGCTTA |  |  |

^a^ Kress WJ, Erickson DL. A two-locus global DNA barcode for land plants: the coding rbcL gene complements the non-coding trnH-psbA spacer region. PLoS ONE. 2007; 2: e508.

^b^ Pacak A, Szweykowska-Kulińska Z. Organellar inheritance in liverworts: an example of *Pellia borealis*. J Mol Evol. 2003; 56: 11-17.

^c^ Sang T, Crawford DJ, Stuessy TF. Chloroplast DNA phylogeny, reticulate evolution and biogeography of *Paeonia* (Paeoniaceae). Am J Bot. 1997; 84: 1120–1136.

^d^ Sawicki J, Plášek V, Szczecińska M Molecular studies resolve *Nyholmiella* (Orthotrichaceae) as a separate genus. J Syst Evol. 2010; 48: 183-194.
