# Supplementary material for: Does Calypogeia azurea (Calypogeiaceae, Marchantiophyta) occur outside Europe? Molecular and morphological evidence
Source: PLoS One. 2018 Oct 10;13(10):e0204561. doi: 10.1371/journal.pone.0204561 (PMC6179228; doi:10.1371/journal.pone.0204561)
Supplement: S4 Table — The means, minimum and maximum values of 47 quantitative traits were calculated according to Buczkowska (2004), N—number of measurements. (PDF) [file pone.0204561.s006.pdf]

S4 Table. Descriptive statistics for C. *azurea* s. str., *C. azurea* – NA, *C. sinensis* and *C. orientalis.*

The means, minimum and maximum values of 47 quantitative traits were calculated according to Buczkowska (2004), N - number of measurements.

		C. azurea s. str. N = 800			*C. azurea - NA* N = 300			*C. sinensis* N = 150			*C. orientalis* N = 450		
No.	**Character**	Mean	Min	Max	Mean	Min	Max	Mean	Min	Max	Mean	Min	Max
1	width of cells of rhizoid initial field in underleaf	21,31	19,99	23,86	16,52	15,17	18,45	13,92	12,91	14,92	13,11	12,20	14,18
2	length of cells of rhizoid initial field in underleaf	23,70	20,47	28,30	19,36	16,52	20,90	18,22	16,70	19,73	14,70	13,25	16,43
3	width of cells in underleaf lobe	33,68	27,80	38,84	27,93	24,86	33,60	25,27	24,40	26,14	23,03	20,33	27,43
4	length of cells in underleaf lobe	50,19	44,17	61,02	39,59	36,12	47,00	40,24	36,79	43,68	29,76	26,90	32,43
5	width of cells in underleaf middle	30,37	25,45	38,52	27,23	23,77	31,24	23,10	22,32	23,88	22,26	19,42	25,62
6	length of cells in underleaf middle	43,30	36,55	53,63	43,25	36,75	48,39	37,11	35,47	38,75	30,91	25,13	38,24
7	number of cells between the sinus and base of underl.	4,54	3,00	6,00	3,25	2,00	4,00	3,13	2,00	4,00	2,14	1,00	3,00
8	width of underleaf	681,99	485,56	943,34	579,67	303,11	772,51	743,43	406,91	1009,95	458,64	381,37	805,68
9	length of the whole underleaf	586,03	402,22	788,66	519,39	258,54	688,23	571,35	306	556,79	335,74	202,77	301,06
10	length of underleaf to the base of rhizoid initial field	498,19	346,67	642,22	400,67	200,89	582,95	445,42	246,45	430,39	264,35	160,75	301,26
11	underleaf sinus depth	189,09	137,78	264,44	170,46	124,51	229,26	168,21	134,96	201,47	108,48	74,42	155,58
12	ratio of width to height of underleaf - 8/9	1,17	0,96	1,30	1,12	1,05	1,17	1,30	1,30	1,81	1,38	1,71	1,91
13	'measure' of underleaf decurrence - 9/10	1,18	1,13	1,35	1,31	1,18	1,44	1,28	1,22	1,34	1,27	1,20	1,34
14	ratio of width of underleaf to width of stem - 8/47	2,14	1,50	2,84	2,27	1,48	2,76	2,61	2,13	3,09	2,23	1,72	2,67
15	width of marginal cells in dorsal part of leaf	38,81	35,13	43,22	31,00	28,93	32,18	33,89	32,42	35,36	34,48	28,09	44,15
16	length of marginal cells in dorsal part of leaf	31,28	29,04	34,65	27,93	23,10	33,56	23,21	21,27	25,16	24,71	19,67	34,93
17	width of median cells in leaf	41,92	34,46	46,36	30,70	27,65	34,41	26,39	24,19	28,59	33,87	27,30	42,92
18	length of median cells in leaf	50,00	43,89	60,35	37,61	33,70	45,00	36,23	35,84	36,61	40,96	31,52	51,23
19	width of cells at ventral leaf base	40,69	34,34	48,36	31,78	31,17	32,35	25,30	23,20	27,39	32,24	27,20	40,51
20	length of cells at ventral leaf base	61,26	52,65	73,02	49,28	46,04	52,51	48,22	47,98	48,46	46,97	34,18	58,91
21	width of 2nd row marginal cells in dorsal part of leaf	36,30	30,84	39,70	29,55	23,28	34,68	26,02	25,86	26,18	27,55	23,76	35,67
22	length of 2nd row marginal cells in dorsal part of leaf	38,45	35,61	44,36	32,31	28,11	37,96	30,59	30,54	30,65	32,32	25,91	43,92
23	length of leaf	1206,31	972,22	1621,11	953,77	604,39	1124,16	1325,97	1225,32	1426,62	1044,48	824,12	1469,27
24	width of leaf	1419,41	1093,33	2171,11	1070,61	588,43	1388,96	1185,02	1022,34	1347,71	943,80	718,24	1281,19
25	height of dorsal part of leaf	475,77	357,78	707,94	407,77	255,49	472,17	456,81	383,32	530,30	390,54	296,34	511,82
26	distance from apex to ventral base of leaf	1478,71	1128,89	2067,78	1201,88	741,69	1431,15	1483,54	1473,90	1493,17	1117,60	862,08	1511,96
27	length of the 1st coordinate	1052,24	811,11	1576,66	845,39	497,23	1022,79	1041,34	875,09	1207,60	846,31	657,84	1191,78
28	length of the 2nd coordinate	1008,82	753,33	1547,77	798,03	457,68	1003,97	946,41	778,89	1113,93	757,42	583,41	1069,37
29	length of the 3rd coordinate	965,20	654,44	1523,33	734,46	392,58	959,05	860,17	674,13	1046,20	684,86	521,03	976,66
30	ratio of length to width of leaf - 23/24	0,87	0,75	0,96	0,91	0,77	1,03	1,13	1,06	1,20	1,11	1,03	1,16
31	ratio of leaf length to distance from apex to ventral leaf base - 23/26	0,82	0,73	0,90	0,80	0,78	0,81	0,89	0,83	0,96	0,93	0,87	0,97
32	ratio of distance A-C to the 1st coordinate - 26/27	1,42	1,30	1,49	1,43	1,33	1,49	1,46	1,24	1,68	1,33	1,27	1,40
33	ratio of length of leaf dorsal part to width of leaf 25/24	0,34	0,30	0,36	0,39	0,34	0,43	0,38	0,37	0,39	0,42	0,40	0,45
34	width of stem cells in the 4th internode	27,71	20,05	34,83	21,20	15,13	28,08	23,06	22,71	23,41	19,95	18,48	22,07
35	length of stem cells in the 4th internode	59,46	46,30	86,87	57,21	30,46	76,54	55,65	50,64	60,65	50,41	41,05	55,68
36	width of stem cells in the 5th internode	27,71	20,05	35,05	21,20	15,13	28,08	23,06	22,71	23,41	19,95	18,48	22,07
37	length of stem cells in the 5th internode	60,08	41,63	89,21	57,21	30,46	76,54	55,65	50,64	60,65	50,41	41,05	55,68

38	width of stem cells in the 6th internode	27,72	20,05	34,83	21,20	15,13	28,08	23,06	22,71	23,41	19,95	18,48	22,07
39	length of stem cells in the 6th internode	60,68	47,79	84,11	57,21	30,46	76,54	55,65	50,64	60,65	50,41	41,05	55,68
40	number of stem cells in the 4th internode	10,10	6,93	13,93	9,25	8,00	12,00	10,50	10,00	11,00	8,14	7,00	10,00
41	number of stem cells in the 5th internode	10,15	6,74	14,87	11,00	10,00	13,00	10,00	9,00	11,00	9,71	8,00	11,00
42	number of stem cells in the 6th internode	10,47	6,93	14,53	12,25	11,00	14,00	11,00	10,00	12,00	10,36	8,00	11,00
43	length of the 4th internode	707,98	536,98	1171,58	578,12	312,37	760,33	719,45	632,54	806,35	435,94	323,46	552,56
44	length of the 5th internode	719,42	518,67	1250,91	578,12	312,37	760,33	719,45	632,54	806,35	435,94	323,46	552,56
45	length of the 6th internode	731,63	500,36	1250,91	578,12	312,37	760,33	719,45	632,54	806,35	435,94	323,46	552,56
46	width of the whole plant	1993,24	1697,58	2773,97	1678,12	1239,86	2071,87	1939,36	1927,25	2951,46	1597,71	1291,28	2436,43
47	width of stem (without leaves)	330,17	266,81	453,99	251,35	204,20	301,05	295,53	235,07	355,99	206,28	166,15	238,03
