# Supplementary material for: Does Calypogeia azurea (Calypogeiaceae, Marchantiophyta) occur outside Europe? Molecular and morphological evidence
Source: PLoS One. 2018 Oct 10;13(10):e0204561. doi: 10.1371/journal.pone.0204561 (PMC6179228; doi:10.1371/journal.pone.0204561)
Supplement: S1 Appendix — (PDF) [file pone.0204561.s007.pdf]

S1 Appendix. The key to *Calypogeia* with blue oil bodies

1. Underleaves as large as leaves or somewhat smaller, transversely elliptic, with a round to shallow emarginate apex …………………………………………………….. ***C. aeruginosa***

1. Underleaves smaller than 1/2-1/3 of leaf size, rounded to obtrapezoidal, deeply bilobed to bisbifid ………………………………………………………………………….……….… **2**

2. Underleaves bifid, with rounded lateral margins or with small additional teeth on one or each lateral side …………………….………………………………………………… **3**

2. Underleaves bisbifid …………………………………………………………………… **4**

3. Undivided area between underleaf sinus bottom and rhizoid initial cells 5-6 cells high, oil bodies botryoidal, deep blue [boreal to temperate Europe and North America] ….… ***C. azurea***

3. Undivided area between the bottom of the underleaf sinus and rhizoid initial cells with 2-3 cells high, oil bodies coarsely granular, deep blue to blue brown [a meta-Himalayan subtropical taxon currently known in southern China and North Vietnam] ….…… ***C. sinensis***

4. Leaves acute, not bifid ………………………………………………………..… **5**

4. Leaves commonly bifid, rarely acute …………………………………………………… **6**

5. Oil bodies coarsely granular, plants flaccid, brow to yellow-brown [meta-Himalaya to Indochina] ……………………………………………………………………… ***C. lunata***

5. Oil bodies botryoidal, plants not flaccid (merely rigid), greenish to bluegreen [temperate amphi-Pacific East Asia] …………………………...………………………..… ***C. orientalis***

6. Oil bodies botryoidal and deep blue … [temperate amphi-Pacific East Asia] … ***C. orientalis***

6. Oil bodies coarsely granular, deep blue to gray and brown ……………………………….. **7**

7. Oil bodies finely granular, blue to brown and gray, with color variation in the same leaf [temperate amphi-Pacific East Asia, extending south-west as far as the Chinese province of Guizhou] …………………………………………………….………………… ***C. granulata***

7. Oil bodies coarsely granular, deep blue throughout [tropical areas or temperate North America] ………………………………………………………………………………… **8***

8. [South-East Asia extending eastward to Samoa] ……….……….…………. ***C. goebelii***

8. [New World: eastern North America and Neotropics] ……………...……...… ***C. peruviana***

* - the relationships between the two taxa keyed out below are unclear, we did not have sufficient material arrive at reliable conclusions.
